# Enhancing Anti‐Tumor Effects of Engineered Extracellular Vesicles via Endocytosis Route Switching and Interferon Response Suppression

**DOI:** 10.1002/advs.202515472

**Published:** 2025-09-23

**Authors:** Zixuan Huang, Chaoqun Lu, Yixin Wang, Huajian Xian, Yuling Zheng, Ting Kang, Rufang Xiang, Shufeng Xie, Minghui Wang, Zeyi Li, Xiaoli Xia, Yaoyifu Yu, Wenjie Zhang, Huijian Zheng, Renyao Pan, Dan Li, Chunjun Zhao, Han Liu

**Affiliations:** ^1^ Shanghai Institute of Hematology State Key Laboratory of Medical Genomics National Research Center for Translational Medicine at Shanghai Ruijin Hospital Shanghai Jiao Tong University School of Medicine and School of Life Sciences and Biotechnology Shanghai 200025 China; ^2^ Department of Oncology Xin Hua Hospital Shanghai Jiao Tong University School of Medicine Shanghai 200092 China; ^3^ Department of General Practice Ruijin Hospital Shanghai Jiao Tong University School of Medicine Shanghai 200025 China; ^4^ Fujian Institute of Hematology Fujian Provincial Key Laboratory on Hematology Fujian Medical University Union Hospital Fuzhou 350001 China

**Keywords:** B‐ALL, cellular therapy, Exosome, Extracellular vesicles, Microvesicles

## Abstract

Engineered extracellular vesicles (EVs) represent a promising therapeutic strategy with many applications in cancer therapy. EVs derived from engineered tumor‐targeting killer cells, such as chimeric antigen receptor (CAR)‐T cells. However, the application of CAR‐T‐EVs is limited by several drawbacks. This study shows that engineered EVs with potent cancer‐targeting and killing abilities can be generated from easily manipulable non‐killer cells, providing a solution to overcome the limitations of CAR‐T‐EVs. It is found that EVs derived from non‐killer cells such as CD19‐targeting 293 cells possess target cell killing capacities comparable to those derived from CD19‐CAR‐T cells. A technique is developed to ensure the presence of sufficient targeting modules on the EV surface using a chimeric CD8‐CD63/CD81 transmembrane region. Uptake of CD19‐targeting EVs by target cells can be optimized by switching the route of CD19 endocytosis from clathrin‐mediated endocytosis (CME) to aggregation‐dependent endocytosis (ADE), leading to lysosomal degradation of the CD19/EVs complex. Degradation of the EVs leads to impairment in the IFN response and subsequent enhancement in EV uptake by target cells, creating a potent feedback cycle. CD19 depletion results in the disruption of the CD19‐AKT‐Myc pathway in the target cells, enhancing the killing capacity both in vitro and in vivo.

## Graphical abstract

CD19‐293‐EV can switch the CD19 endocytosis pathway from clathrin‐mediated endocytosis to aggregation‐dependent endocytosis, leading to lysosomal degradation of the CD19/EVs complex. Degradation of the EVs leads to impairment in the IFN response and enhancement in EV uptake by target cells, creating a potent feedback cycle. CD19 depletion leads to disruption of the CD19‐AKT‐Myc pathway in target cells.

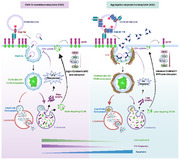

## 1 Introduction

Extracellular vesicles (EVs) are small sacs enveloped by a cell‐derived lipid bilayer membrane that are categorized into several subtypes, including exosomes (EXOs) and microvesicles (MVs). EVs play a crucial role in intercellular communication and are intricately involved in various cellular processes.^[^ ^1^ ^]^ Their small size allows them to navigate effortlessly within the tumor environment, facilitating the rapid delivery of target molecules to specific tissues.^[^ ^2^, ^3^, ^4^ ^]^ As a result, the development of EV‐based therapies represents a transformative advancement in precision cancer therapy.

Natural EVs possess inherent potential for treating diseases. For example, mesenchymal stem cell‐derived EXOs have been shown to ameliorate hypoxic pulmonary hypertension.^[^ ^5^ ^]^ However, the application of natural EVs in cancer therapy is hindered by their limited tumor‐killing efficacy and insufficient targeting specificity. To overcome these limitations, natural EVs can be engineered to improve their tumor‐targeting capabilities and cytotoxic potential. Engineered EVs can perform diverse functions, including targeted delivery of chemotherapeutic agents, modulation of gene expression in cancer cells, and promotion of anti‐tumor immune responses, and they can be used in combination with various other therapeutic strategies.^[^ ^6^, ^7^, ^8^ ^]^

Engineered EVs can be generated by either modifying natural EVs or by harvesting them from engineered cells.^[^ ^9^, ^10^, ^11^, ^12^, ^13^ ^]^ The targeting capability of natural EVs can be enhanced through drug loading and surface modifications, including the incorporation of nanobodies, tumor‐targeting peptides, nucleic acid aptamers, and superparamagnetic nanoparticles. EVs can be used for drug delivery due to their structural and functional similarities to synthetic drug carriers like liposomes.^[^ ^14^, ^15^, ^16^ ^]^ By loading EVs with small molecules, proteins, or nucleic acid‐based anticancer agents, their tumor‐killing efficacy can also be substantially enhanced.^[^ ^17^, ^18^, ^19^, ^20^, ^21^ ^]^ However, the process of loading targeting molecules or drugs onto EVs presents several challenges. The loading procedure is often technically complex, with low efficiency and potential risk of damaging the structural integrity of the EV. These limitations hinder their universal application in clinical settings.^[^ ^22^, ^23^ ^]^

Extraction from engineered cancer‐targeting killer cells, such as chimeric antigen receptor (CAR)‐T and CAR‐NK, represents an important alternative production strategy for engineered EVs.^[^ ^24^, ^25^ ^]^ CAR‐T cells, for instance, release EXOs with CAR on their surface, and equipping these CAR‐loaded EXOs with a variety of cytotoxic molecules enables them to function as potent tumor‐targeting agents. The tumor‐killing efficacy of T cell‐derived EVs is attributed to lethal compounds such as granzymes, lysosomal enzymes, and perforin.^[^ ^26^, ^27^ ^]^ It is therefore plausible that EVs derived from CAR‐T cells could serve as highly effective eliminators of cancer target cells. However, the production of CAR‐T cells is a complex process, requiring advanced culturing techniques and substantial investments of both time and resources. Furthermore, CAR‐T cells have limited expansion potential and EV yields, making it difficult to obtain large quantities of EVs in vitro. CAR‐T‐EV therapy also carries the risk of inducing cytokine release syndrome (CRS), a potentially life‐threatening inflammatory response triggered by the activation of immune cells.^[^ ^28^, ^29^ ^]^

Producing EVs from immortalized non‐killer cells, such as HEK293 cells, offers several advantages, including high yield, simplified and standardized production procedures, enhanced safety, reduced immunogenicity, and no CRS‐risk. The HEK293 cell line is particularly well‐suited for EV production due to its high transfection efficiency,^[^ ^30^, ^31^ ^]^ rapid growth rate, and high EV yield.^[^ ^32^, ^33^, ^34^ ^]^ Moreover, HEK293‐derived exosomes have low in vivo toxicity and immunogenicity.^[^ ^35^ ^]^ Given these advantages, the generation of engineered EVs with robust tumor‐targeting and killing capabilities from immortalized non‐killer cells would be expected to circumvent the challenges faced by CAR‐T‐EVs. However, EVs derived from immortalized non‐killer cells often require sophisticated modifications to achieve sufficient tumor‐targeting and anti‐tumor activity. Thus, it is imperative that more direct and effective methods of modifying EVs are developed.

In this study, we provide proof‐of‐principle for a simple and efficient strategy to generate therapeutic EVs from engineered non‐killer cells. We demonstrate that this strategy can be used to generate engineered EVs with high cancer‐targeting and killing capacity from engineered non‐killer cells expressing targeting modules. Though these EVs are derived from non‐killer cells, they are capable of killing target cells through utilization of an underappreciated killing mechanism involving the endocytosis and depletion of target antigens. We also show that expressing targeting modules on the EV surface using a chimeric CD8‐CD63/CD81 transmembrane domain suppresses interferon (IFN) response, further enhancing anti‐tumor capability.

## 2 Results

### 2.1 CD19‐293‐EV has Superior Target Cell Killing Efficacy Compared to CD19‐CAR‐T‐EV

**Figure 1:**
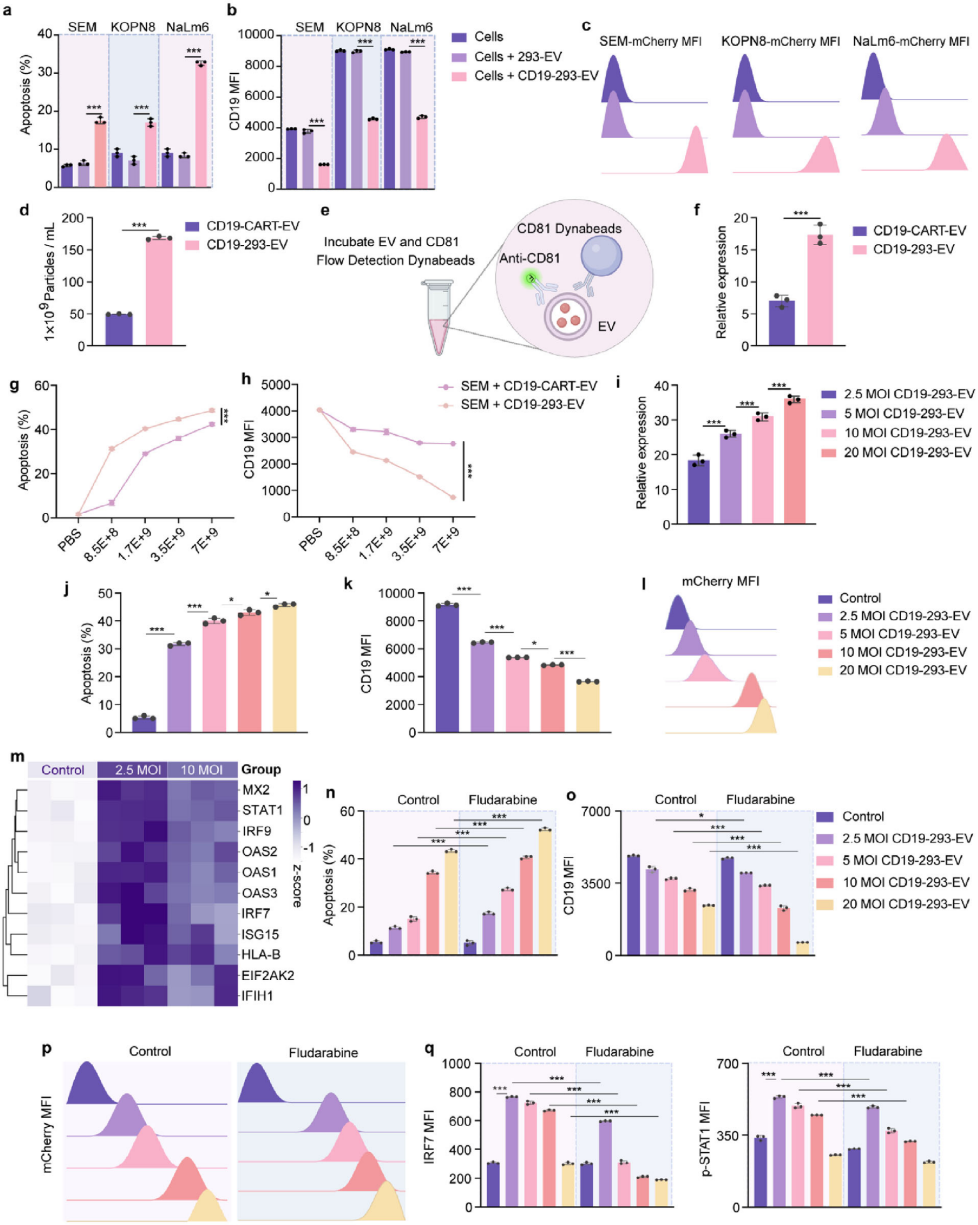
Increased abundance of targeting modules on CD19‐293‐EVs enhances anti‐tumor capacity by inhibiting interferon response. a–c) SEM, KOPN8, and NaLm6 cells were co‐cultured with 293 and CD19‐293 cells for 24 h and then assessed by flow cytometry for (a) target cell apoptosis, (b) CD19 MFI, and (c) EV uptake level. The ratio of effector cells to target cells (E:T) was 5:1. 293‐EV as a negative control. d) NTA calculation of EV production for the same number of cells. e) Flow diagram of CD81‐dynabeads‐EV detection. EVs were adhered to CD81‐dynabeads and then incubated with CD81 antibody, allowing for the determination of the average fluorescence of EVs by flow cytometry. f) Relative expression of targeting modules on EV as a percentage of EV‐expressed CD81. g,h) SEM cells were treated with EVs of different particle numbers for 24 h. (g) Target cell apoptosis and (h) CD19 MFI were detected by flow cytometry. i) Relative expression of targeting modules at 2.5, 5, 10, and 20 MOI CD19‐293‐EV. j–l) SEM cells were treated with EVs derived from different MOI CD19‐293 for 24 h and then assessed by flow cytometry for (j) target cell apoptosis, (k) CD19 MFI, and (l) EV uptake. m) Bulk RNA sequencing analysis of SEM cells treated with 2.5 and 10 MOI CD19‐293‐EV for 24 h revealed significant differences in the expression of interferon response pathway‐related genes (*p*<0.05). The results are visualized as a heatmap. n–q) SEM cells were treated with 2.5, 5, 10, and 20 MOI CD19‐293‐EV with or without fludarabine (1 µM) for 24 h and then assessed by flow cytometry for (n) target cell apoptosis, (o) CD19 MFI, (p) EV uptake, and (q) IRF‐7 and p‐STAT1 MFI. PBS was used as a control. The representative result of three independent experiments is shown. Each data point represents the means ± SD (*n* = 3). Statistical analysis was performed using Student's *t*‐test for the unpaired data. Statistical significance: *** *p *< 0.001.

Our previous studies have shown that non‐killer cell‐derived CD19‐targeting effector cells can cause target cell death.^[^ ^36^ ^]^ Consistent with these findings, we found that CD19‐targeting HEK 293 (CD19‐293) cells expressing a CD19‐targeting module (CD19‐TM) consisting of a CD19‐specific scFv and a CD8 hinge/transmembrane domain (Figure S1, Supporting Information) effectively deplete CD19 on B‐ALL target cells and induce target cell death^[^ ^36^ ^]^ (Figure S2, Supporting Information). We also confirmed through compartmentalized co‐culture of CD19‐293 and ALL cells (SEM cells) in Transwell plates that CD19‐293 cells exert their killing effect in a cell‐to‐cell contact‐independent manner (Figure S3, Supporting Information). Since 293 cells are non‐killer cells and are therefore incapable of secreting cytotoxic cytokines, we hypothesized that the observed contact‐independent cytotoxicity may be mediated by EVs derived from CD19‐293 cells (CD19‐293‐EVs). To test this hypothesis, B‐ALL cells were incubated with CD19‐293‐EVs extracted via ultrafiltration. CD19‐293‐EV treatment induced CD19 depletion in target B‐ALL cells and exhibited direct cytotoxic effects (**Figure** **1**a,b). The specificity of CD19‐EV was demonstrated using 293‐EV as a control, and off‐target effects were excluded. Moreover, CD19‐293‐EVs were efficiently enriched on target cells (Figure 1c).

Our previous findings indicated that CD19‐targeting effector cells induce CD19 antigen depletion on B‐ALL target cells, which undermines the survival of target cells via disruption of the CD19/AKT/MYC axis.^[^ ^36^ ^]^ We found that the expression of both the PI3K/AKT pathway and MYC were compromised in SEM cells as a result of CD19‐293‐EV‐mediated CD19 depletion. Indeed, CD19‐293‐EV treatment induces the downregulation of CD19, p‐AKT, and MYC, indicating that, like CD19‐293, CD19‐293‐EVs also trigger B‐ALL target cell death via disruption of the CD19/AKT/MYC axis (Figure S4, Supporting Information). Taken together, these results suggest that CD19‐293 cell‐induced target cell death is at least partially mediated by CD19‐293‐EVs, making them a promising potential tool for cell therapy.

To compare their cytotoxic potential, CD19‐293 and CD19‐CAR‐T cells were co‐cultured with B‐ALL cells to evaluate their killing efficiency (Figure S5, Supporting Information). Notably, ≈50‐fold more CD19‐293 cells were required to achieve similar SEM cell killing outcomes compared to CD19‐CAR‐T cells (Figure 1a; Figure S5, Supporting Information), indicating that the cytotoxic capacity of CD19‐293 cells was significantly lower than that of CD19‐CAR‐T cells.

To characterize CD19‐CART‐EV and CD19‐293‐EV, both were extracted from cell cultures via ultrafiltration and subjected to transmission electron microscopy (TEM)^[^ ^37^ ^]^ (Figure S6, Supporting Information). CD19‐CART‐EV and CD19‐293‐EV had an internally hollow round or oval morphology with clearly visible bilayer lipid membranes (Figure S6, Supporting Information). To facilitate direct comparisons between vesicle subsets, we normalized EV doses by particle count, as measured by nanoparticle tracking analysis (NTA)^[^ ^38^ ^]^ (Figure S7, Supporting Information). The EV yield of CD19‐293 cells was significantly higher than that of CD19‐CAR‐T cells (Figure 1d). To compare the abundance of the CD19‐targeting module between CD19‐293‐EVs and CD19‐CAR‐T‐EVs, EVs were adsorbed to CD81‐dynabeads and analyzed by flow cytometry^[^ ^39^ ^]^ (Figure 1e). The abundance of CD19‐targeting modules was significantly higher on CD19‐293‐EVs than on CD19‐CAR‐T‐EVs (Figure 1f).

We then compared the killing ability of CD19‐293‐EVs and CD19‐CAR‐T‐EVs against B‐ALL target cells. At the same number of EV particles, CD19‐293‐EV exhibited a stronger killing effect (Figure 1g; Figure S8a, Supporting Information), leading to more pronounced CD19 depletion (Figure 1h; Figure S8b, Supporting Information) and higher EV enrichment on target cells (Figure S8c,d, Supporting Information). These results suggest that, despite the significantly weaker cytotoxicity of CD19‐293 cells compared to CD19‐CAR‐T cells, CD19‐293‐EV is superior to CD19‐CAR‐T‐EV at effectively killing target cells. This contradiction may be explained by the fact that CAR‐T cells primarily exert their cytotoxic effects through the potent Cytotoxic T Lymphocyte (CTL) mechanism that relies on direct cell‐to‐cell contact rather than on contact‐independent CAR‐T‐EV‐mediated killing.

### 2.2 Increased Abundance of Targeting Modules Enhances EV Cytotoxicity

We next explored the mechanism underlying the superior killing ability of CD19‐293‐EV compared to CD19‐CAR‐T‐EV despite the presence of cytotoxic molecules such as perforin in the latter.^[^ ^25^, ^27^ ^]^ We hypothesized that the enhanced cytotoxicity of CD19‐293‐EV could be attributed to the increased abundance of CD19‐targeting modules on CD19‐293‐EVs. To test this hypothesis, we generated CD19‐293 cells with varying multiplicities of infection (MOIs) to produce EVs with different abundances of CD19‐targeting modules (Figure 1i). These EVs were subsequently characterized and quantified using TEM (Figure S9, Supporting Information) and NTA (Figure S10, Supporting Information), and their characteristics (e.g., morphology and particle size) met the criteria for EVs.

To determine whether an increase in the abundance of CD19‐targeting modules on EVs enhances their killing ability, we compared the cytotoxic effects of EVs with different levels of CD19‐targeting modules. SEM and KOPN8 cells treated with CD19‐293‐EVs with increased abundance of CD19‐targeting modules demonstrated a dose‐dependent increase in tumor cell killing capability (Figure 1j; Figure S11a, Supporting Information), CD19 depletion (Figure 1k; Figure S11b, Supporting Information), and EV uptake (Figure 1l; Figure S11c, Supporting Information). Taken together, these findings indicate that increasing the abundance of targeting modules on EVs enhances their killing ability.

### 2.3 Inhibition of IFN Response Enhances the Efficacy of EV‐Mediated Target Cell Killing

To investigate the mechanism by which increased abundance of targeting modules on EVs enhances their killing capacity, we performed RNA‐seq analysis on SEM cells treated with CD19‐293‐EV. The results revealed that IFN response‐related genes were significantly upregulated upon treatment with CD19‐293‐EVs (Figure S12, Supporting Information). However, increasing the abundance of the targeting modules on EVs gradually attenuated the IFN response (Figure 1m). These results suggest that the IFN response plays an inhibitory role in EV‐mediated cytotoxicity and that increases in the abundance of targeting modules on EVs enhance their killing ability by attenuating the IFN response.

To validate the inhibitory role of the IFN response, we tested whether EV‐mediated cytotoxicity can be enhanced by fludarabine, a STAT1 inhibitor that plays a crucial role in IFN response.^[^ ^40^, ^41^, ^42^ ^]^ We compared the effects of treatment with EVs with different abundances of targeting modules on target cells in the presence and absence of fludarabine, with a specific focus on IRF7, a major target of the IFN response and STAT1.^[^ ^43^ ^]^ Treatment with fludarabine markedly enhanced EV‐mediated cytotoxicity and CD19 depletion (Figure 1m–o; Figure S13a,b, Supporting Information), which was associated with an increase in EV uptake by target cells (Figure 1p; Figure S13c, Supporting Information). Fludarabine, when used alone, demonstrated minimal to no effect and was comparable to the control group. Moreover, fludarabine treatment led to a significant reduction in IRF7 and p‐STAT1 expression in SEM cells, indicating that fludarabine enhances EV‐mediated cytotoxicity through inhibition of the IFN response (Figure 1q; Figure S13d,e, Supporting Information). Together, these results suggest that the IFN response inhibits EV‐mediated cytotoxicity and CD19 depletion through preventing the uptake of EVs by target cells.

### 2.4 The Abundance of Targeting Modules on EVs can be Increased through Structural Optimization

**Figure 2:**
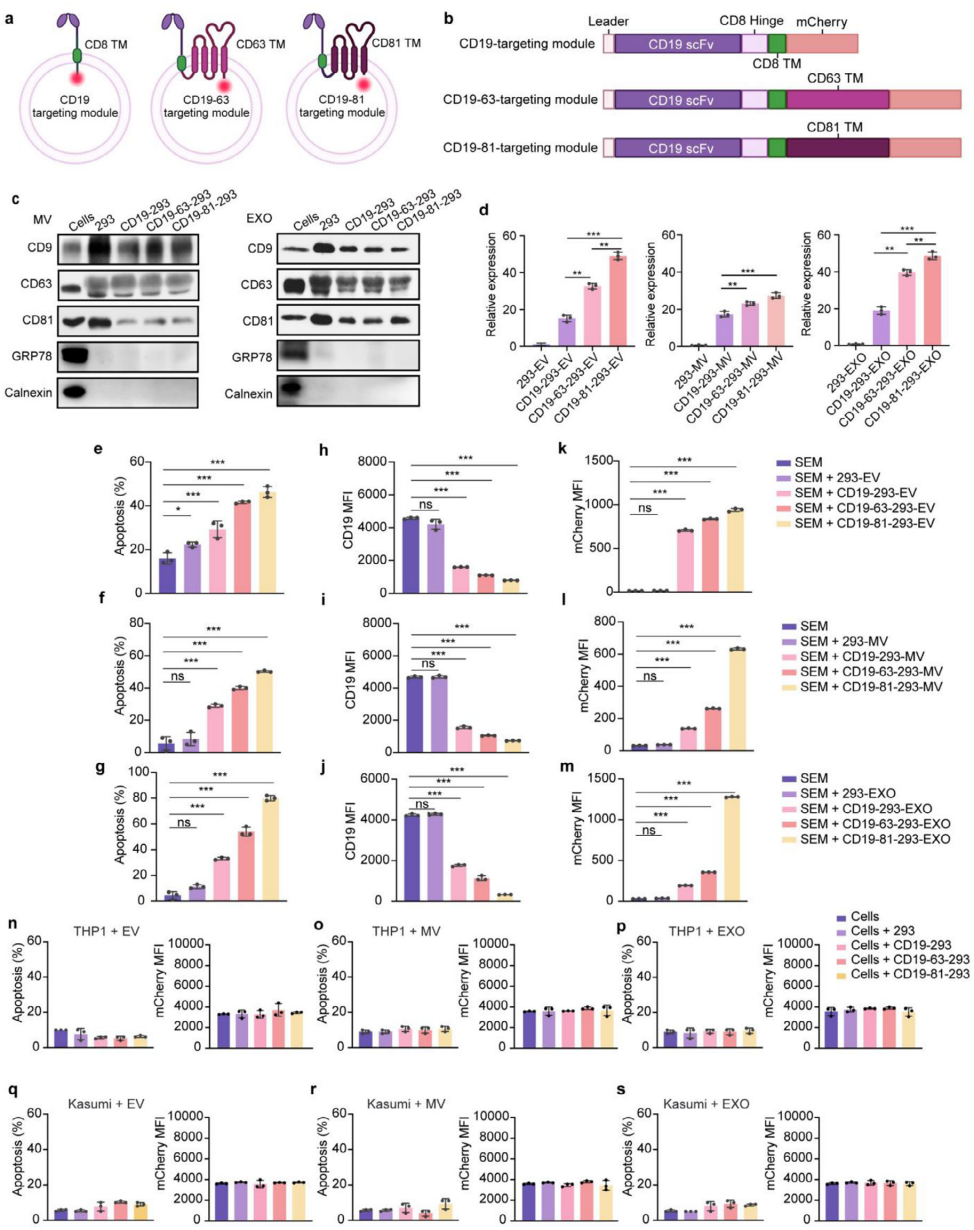
B‐ALL cells are destroyed by engineered EVs. a,b) Diagram and (b) plasmid mapping of CD19‐293, CD19‐63‐293, and CD19‐81‐293 cells. c) Western blot analysis of CD9, CD63, CD81, GRP78, and Calnexin expression on the surface of MVs and EXOs. d) Flow cytometric analysis of the relative expression levels of CD81 and targeting modules on EVs, MVs, and EXOs produced under specific conditions. e–g) Target cell apoptosis in SEM cells after 48 h treatment with (e) EVs, (f) MVs, and (g) EXOs. h–j) CD19 MFI in SEM after 48 h treatment with (h) EVs, (i) MVs, and (j) EXOs. k–m) Vesicle uptake levels in SEM cells after 48 h treatment with (k) EVs, (l) MVs, and (m) EXOs. n–s) Detection of apoptosis and vesicle uptake levels in (n‐p) THP1 and (q‐s) Kasumi‐1 cells after 24 h treatment with (n, q) EVs, (o, r) MVs, and (p,s) EXOs. mCherry‐MFI was used to determine vesicle uptake levels. 293‐EV as a negative control. The representative result of three independent experiments is shown. Each data point represents the means ± SD (*n* = 3). Statistical analysis was performed using Student's *t*‐test for the unpaired data. Statistical significance: *** *p *< 0.001. Image created with BioRender.com, used with permission.

We investigated whether structural optimization of the targeting modules could increase the number of the targeting modules on EVs. Given that the EV membrane is enriched in CD63 and CD81, we hypothesized that anchoring the targeting module to the EV surface through the transmembrane domains of CD63 and CD81 would enhance EV surface presentation of the targeting modules. Since the N‐terminus of the transmembrane domains of CD63 and CD81 lie on the internal side of the membrane, we designed CD8‐CD63/CD8‐CD81 chimeric transmembrane domains to anchor targeting modules to the surface of EVs (**Figure** **2**a,b).

Comparing the killing capacity of CD19‐293, CD19‐63‐293 (with CD19‐scFv‐CD8‐CD63 targeting module), and CD19‐81‐293 (with CD19‐scFv‐CD8‐CD81 targeting module) cells (Figure S14, Supporting Information) revealed that both CD19‐63‐293 and CD19‐81‐293 cells exhibited potent target killing efficacy and efficiently depleted CD19 in B‐ALL cells (Figure S15, Supporting Information), indicating that the function of targeting modules was not compromised by structural optimization. However, some CD19‐positive B cells, such as Raji cells, exhibited resistance to being killed by the engineered cells (Figure S15g,h, Supporting Information), suggesting that these cells do not require CD19/AKT signaling for survival. Interestingly, Raji cells also exhibited poor response to CAR‐T‐mediated killing.^[^ ^36^ ^]^ This cross‐resistance suggests that only CAR‐T‐sensitive target cells can be efficiently eradicated by the engineered cells. Moreover, using Transwell co‐culture assays, we confirmed that CD19‐63‐293 and CD19‐81‐293 cells also killed SEM cells in a contact‐independent manner (Figure S16a, Supporting Information). The supernatant of these modified cells, especially that of CD19‐81‐293 cells, induced more robust killing of target cells than that of CD19‐293 cells (Figure S16b, Supporting Information). These results suggest that CD19‐63‐293 and CD19‐81‐293 cells, like CD19‐293 cells, exert contact‐independent cytotoxicity via EVs.

There are four main types of EVs: EXOs, MVs, ectosomes, and apoptotic bodies.^[^ ^44^ ^]^ Among these EV subtypes, MVs and EXOs are the two main populations utilized in tumor therapy.^[^ ^45^ ^]^ To understand which subtype of EV is more strongly cytotoxic, we isolated total EVs (hereafter referred to as EVs), MVs, and EXOs using ultrafiltration, high‐speed centrifugation, and ultracentrifugation, respectively (Figure S17, Supporting Information). The concentration and size of the various EV particles obtained were then measured using NTA (Figure S18, Supporting Information). The observed sizes of 30–150 nm for EVs and EXOs and 100–1000 nm for MVs are consistent with those reported in the literature.^[^ ^46^, ^47^ ^]^ TEM revealed that EVs and EXOs exhibited a characteristic cup‐shaped structure with well‐defined edges, appearing discoidal in form (Figure S19a,b, Supporting Information). By contrast, MVs appeared as spherical or ellipsoidal structures, single or multi‐compartmental, and enclosed by a bilayer membrane^[^ ^37^, ^48^ ^]^ (Figure S19c, Supporting Information).

EVs have specific markers, such as CD9, CD63, CD81.^[^ ^49^ ^]^ The various MVs and EXOs isolated were enriched in these canonical markers and depleted in the endoplasmic reticulum protein GRP78 and calnexin (Figure 2c).^[^ ^50^ ^]^ These results confirm that the protein expression profiles of EVs, MVs, and EXOs align with the expected criteria.^[^ ^24^ ^]^ Notably, CD19‐81‐293‐EV/MV/EXO exhibited the highest targeting module abundance among all vesicle subtypes (Figure 2d; Figure S20, Supporting Information). These results suggest that the abundance of targeting modules on EVs can be increased by structural optimization, particularly through the use of a CD8/CD81 chimeric transmembrane domain.

### 2.5 Targeting Module‐Optimized EVs Possess Efficient Killing Capability

To determine the tumor‐targeting efficacy and cytotoxicity of the engineered EVs, we treated B‐ALL cells with EVs, MVs, and EXOs and measured their killing ability. When EVs, MVs, and EXOs derived from CD19‐293, CD19‐63‐293, and CD19‐81‐293 cells (hereafter referred to as CD19‐293‐EV/MV/EXO, CD19‐63‐293‐EV/MV/EXO, and CD19‐81‐293‐EV/MV/EXO, respectively) were used, significant apoptosis was observed (Figure 2e–g; Figures S21a–c and S22a–d, Supporting Information). Moreover, target cell CD19 levels were reduced (Figure 2h–j; Figures S21 and S22, Supporting Information) and EV uptake was increased (Figure 2k–m; Figures S21 and S22, Supporting Information). Importantly, this increase in uptake correlates with a more drastic depletion of CD19 on target cells and a greater target killing potency. This was especially true for CD19‐81‐293‐EVs/MV/EXO, which has the highest abundance of targeting modules and the most potent killing effects on target cells. Altogether, these findings indicate a strong correlation between target killing ability and abundance of targeting modules on EVs. Modifications of the transmembrane region in CD19‐63‐293 and CD19‐81‐293 cells to increase targeting module abundance can enhance the cytotoxic efficacy of their derived EVs.

Similar to earlier findings, the engineered EVs were unable to kill Raji cells (Figure S22e,f, Supporting Information), though they caused CD19 depletion (Figure S22k,l, Supporting Information) and were taken up efficiently by target cells (Figure S22q,r, Supporting Information). These findings further underscore the critical role of CD19‐dependency in target cell sensitivity to EV‐mediated killing. To confirm the specificity of the engineered EVs, they were also used to treat CD19‐negative THP1 and Kasumi‐1 cells. The engineered EVs could neither induce cell death in CD19‐negative target cells (Figure 2n–s; Figure S23a–f, Supporting Information) nor be taken up by these cells (Figure 2n–s), suggesting a high degree of specificity for these engineered EVs.

### 2.6 Targeting Module‐Optimized EVs Induce Stronger Lysosome‐Mediated Degradation of CD19

**Figure 3:**
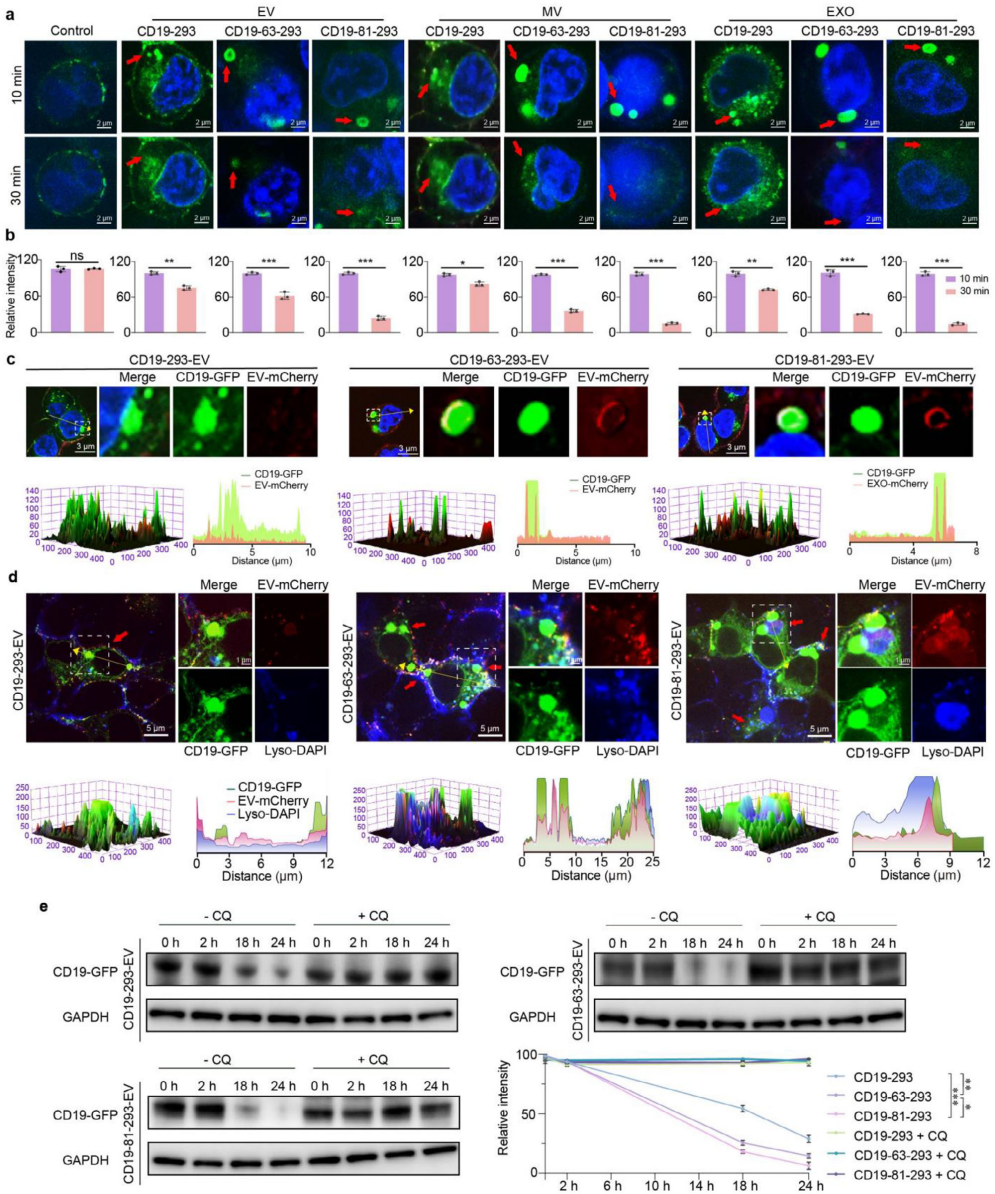
Targeting module‐optimized EVs induce stronger lysosome‐mediated degradation of CD19. a) CD19‐GFP protein expression in 293‐CD19‐GFP cells following treatment with EVs, MVs, and EXOs for 10 and 30 min (blue: Hoechst; green: CD19; red: EVs, MVs, and EXOs). Scale bar: 2 µm. b) Quantitative analysis of CD19‐GFP fluorescence in (a). The relative fluorescent intensity at 10 and 30 min was determined for each group. c) Co‐localization of CD19‐GFP protein and engineered EVs in 293‐CD19‐GFP cells following 10 min treatment with EVs. 3D surface plot of fluorescence levels and corresponding fluorescence curves (blue: Hoechst; green: CD19‐GFP; red: EVs, MVs, and EXOs; yellow: merge). Scale bar: 3 µm. d) Co‐localization of CD19, engineered EVs, and lysosomes in 293‐CD19‐GFP cells following 15 min treatment with engineered EVs. 3D surface plot of fluorescence levels and corresponding fluorescence curves (blue: lysosome; green: CD19; red: EVs; violet: merge). Scale bar; 5 µm. Scale bar of enlarged images: 1 µm. PBS was used as a control. e) Abundance of CD19 protein in 293‐CD19‐GFP cells following treatment with EVs and chloroquine (CQ, 10 µm) for 0, 2, 18, or 24 h, quantified by Western blot analysis. The line chart is the gray analysis of the bands. The representative result of three independent experiments is shown. Each data point represents the means ± SD (*n* = 3). Statistical analysis was performed using Student's *t*‐test for the unpaired data. Statistical significance: *** *p *< 0.001.

To better understand the dynamic between CD19 and EVs in target cells, we performed a time‐lapse analysis in EV‐treated 293 cells expressing CD19‐GFP fusion protein (293‐CD19‐GFP). Compared to the original CD19‐293‐EVs, the targeting module‐optimized EVs resulted in brighter and larger CD19 protein clusters whose CD19‐derived fluorescent signals diminished more rapidly (**Figure** **3**a,b). As predicted by 3D surface plotting, the increased abundance of the targeting module on CD19‐81‐293‐EV/MV/EXO resulted in a large number of EVs (red) clustered around the target cells during treatment of 293‐CD19‐GFP cells. This result suggests that the engineered EVs significantly enhance targeting levels and efficiency. Further analysis revealed that the CD19‐derived fluorescence signal (green) overlapped extensively with the EV‐derived fluorescence signal (red) in CD19‐81‐293‐EV/MV/EXO‐treated 293‐CD19‐GFP cells (Figure 3c; Figure S24a,b, Supporting Information), indicating co‐localization (yellow) of CD19 and the engineered EVs. This result suggests that the engineered EVs more efficiently form complexes with CD19 proteins. Interestingly, the CD19‐GFP‐derived fluorescent signal rapidly diminished within 30 min of EV engagement (Figure 3a,b). Since immunoblot analysis showed that CD19‐GFP proteins were not degraded until 2 h later, this suggests that the CD19‐GFP fusion protein is first denatured, resulting in loss of the fluorescent signal, and then degraded.

While both proteasomes and lysosomes are major sites for intracellular proteolysis, many biomolecular substrates are only appreciably denatured in the acidic milieu of lysosomes. We therefore hypothesized that CD19‐GFP undergoes lysosome‐mediated degradation. To address whether CD19 proteins and EVs are, in fact, degraded in lysosomes, 293‐CD19‐GFP cells were treated with chloroquine (CQ) to prevent lysosome‐mediated denaturation and degradation.^[^ ^51^, ^52^, ^53^ ^]^ Following treatment with the targeting module‐optimized EVs, significant co‐localization was observed between CD19 (green), optimized EVs (red), and lysosomes (blue) (Figure 3d). By contrast, the original CD19‐293‐EV and CD19 only partially co‐localize with lysosomes, and the degree of co‐localization is much lower (Figure 3d). Furthermore, compared to the original CD19‐293‐EVs, the optimized CD19‐293‐EVs induce more rapid and complete depletion of CD19. In all cases, treatment with CQ completely blocks CD19‐293‐EV‐induced degradation of CD19 (Figure 3e). These results indicate that the optimized EVs more efficiently target CD19 to lysosomes, resulting in faster, more complete degradation.

### 2.7 Targeting Module Optimization Shifts CD19 Endocytosis to the Aggregation‐Dependent Pathway

**Figure 4:**
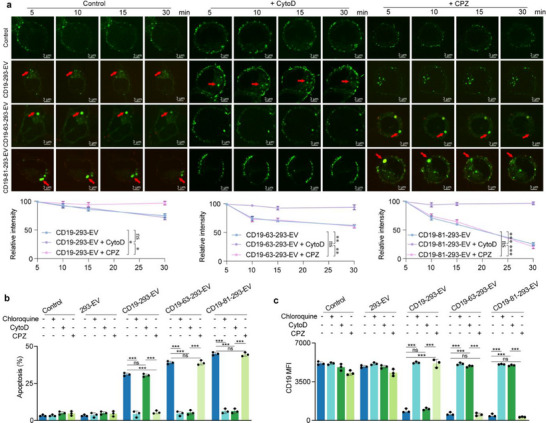
EVs trigger a shift to aggregation‐dependent endocytosis (ADE) of CD19 targeted for lysosomal degradation. a) Expression of CD19 in 293‐CD19‐GFP cells treated with engineered EVs (red) and/or CytoD (10 µm) and chlorpromazine (CPZ, 10 µm) at 5, 10, 15, and 30 min. (blue: DAPI; green: CD19; red: EVs). Scale bar: 3 µm. Quantitative analysis of CD19‐GFP fluorescence based on the relative intensities obtained from the ratio of the 5, 10, 15, and 30 min intensities of each group. b,c) Detection of (b) apoptosis and (c) CD19 MFI in SEM cells following 48 h of treatment with engineered EVs, chloroquine (10 µm), CytoD (10 µm), and CPZ (10 µm). PBS was used as a control. 293‐EV as a negative control. The representative result of three independent experiments is shown. Each data point represents the means ± SD (*n* = 3). Statistical analysis was performed using the Student's *t*‐test for the unpaired data. Statistical significance: *** *p *< 0.001.

The formation of large, aggregated clusters of CD19 in combination with the lysosomal targeting of CD19 suggests that targeting module‐optimized CD19‐293‐EVs trigger a shift to aggregation‐dependent endocytosis (ADE).^[^ ^54^ ^]^ ADE is an actin‐driven endocytic process that resembles macropinocytosis. After protein aggregation occurs, cargoes are redirected into the lysosome, promoting the degradation of surface protein aggregates. To determine whether CD19 endocytosis occurs via the ADE pathway, we blocked ADE by treating 293‐CD19‐GFP cells with CytochalasinD (CytoD), an actin polymerization inhibitor. As expected, CytoD treatment resulted in retention of CD19 on the surface of 293 cells treated with CD19‐63‐293‐EV and CD19‐81‐293‐EV without aggregation or endocytosis (**Figure** **4**a). These results indicate that optimized CD19‐293‐EVs induce CD19 aggregation and endocytosis via the ADE pathway.

Clathrin‐mediated endocytosis (CME) depends on dynamin to scission endocytic carriers, usually resulting in smaller vesicles due to constraints imposed by their coat.^[^ ^54^ ^]^ Since the CD19 signal in the original CD19‐293‐EV followed a small, scattered dot pattern and was not affected by CytoD treatment (Figure 4a), we hypothesized that CME is the main endocytosis route for these EVs. Treatment of 293‐CD19‐GFP cells with the CME inhibitor chlorpromazine (CPZ) blocked original CD19‐293‐EV‐induced endocytosis but not optimized CD19‐293‐EV‐induced endocytosis. These results suggest that targeting module‐optimization of EVs shifts CD19/EV endocytosis from CME to ADE.

We next addressed whether targeting module optimization of EVs shifts the endocytosis pathway of CD19/EV in B‐ALL target cells and how different endocytic routes impact EV‐mediated antigen depletion and cytotoxicity. CytoD and CPZ treatment completely blocked target cell killing, CD19 endocytosis, and EV uptake induced by both the optimized CD19‐293‐EVs and the original CD19‐293‐EVs (Figure 4b,c; Figure S25, Supporting Information). These results demonstrate that targeting module optimization of EVs shifts the CD19/EV endocytic pathway from CME to ADE in B‐ALL target cells, which is critical for EV‐mediated CD19 depletion and target cell killing.

To determine whether the engineered EVs induce lysosomal degradation of CD19 in B‐ALL target cells, as it did in 293‐CD19‐GFP cells, we treated SEM cells with CQ. This resulted in the complete abrogation of EV‐mediated cytotoxicity and degradation of CD19/EV (Figure 4b,c; Figure S25, Supporting Information), indicating that the EV/CD19 complexes are also ultimately targeted to the lysosomes for degradation in B‐ALL target cells. Together, these results demonstrate that CD19‐targeting EV‐mediated cytotoxicity is tightly associated with the depletion of CD19 in target cells and, consequently, the endocytic pathway used.

### 2.8 Targeting Module‐Optimized EVs More Effectively Inhibit the IFN Response and Disrupt the CD19/AKT/MYC Axis

**Figure 5:**
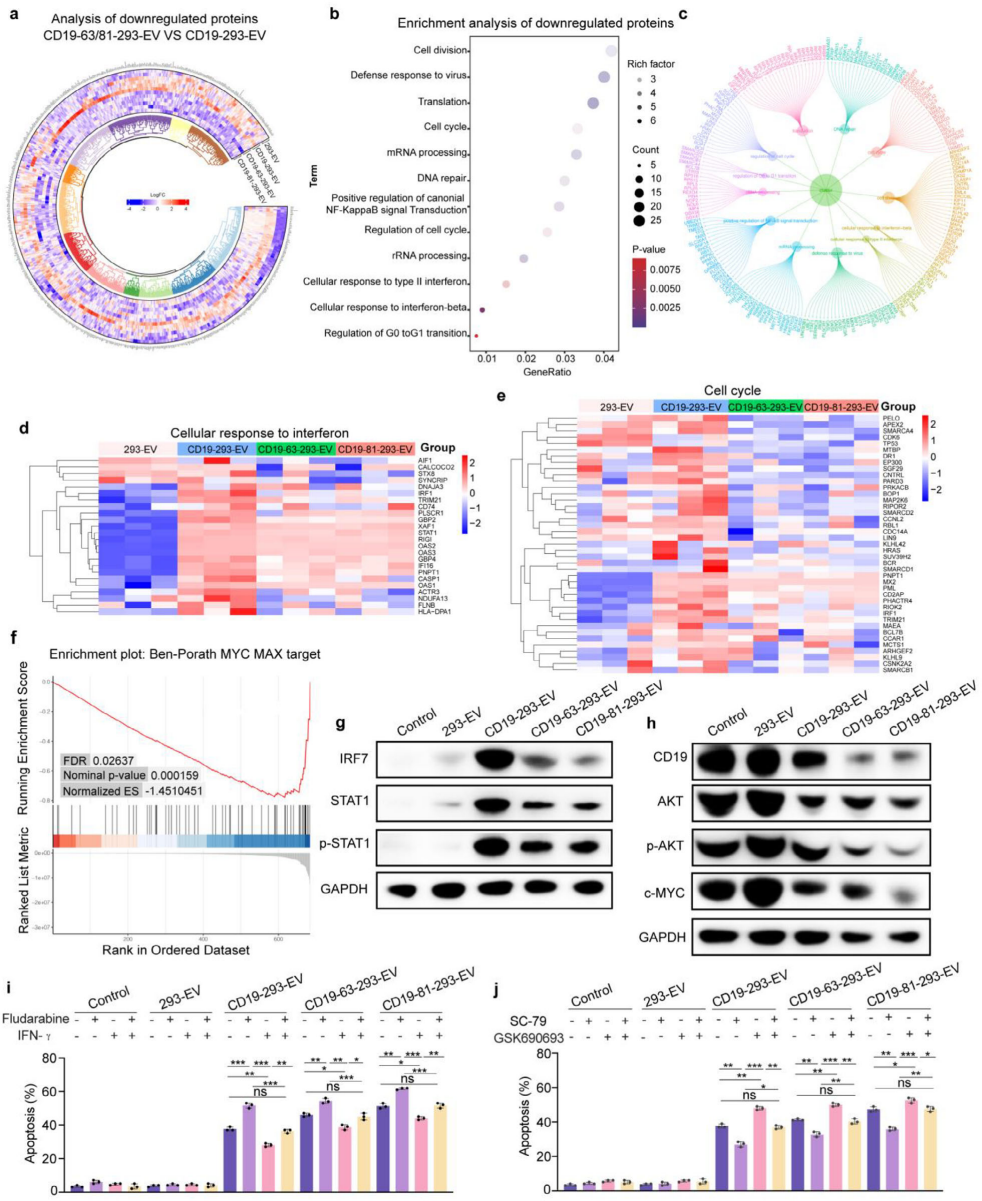
Inhibition of the interferon response and the CD19/AKT/MYC axis is crucial for engineered EV‐mediated killing of B‐ALL cells. a) Proteomics analysis. Heatmap indicating proteins downregulated in SEM cells treated with CD19‐63‐293‐EV and CD19‐81‐293‐EV compared to those treated with CD19‐293‐EV. Each group includes three biological replicates. Differential expression was determined using a significance level of *p*<0.05 and a fold‐change threshold of 2. b) Enrichment analysis. GO terms in biological process (BP) subgroups showing the downregulated proteins. c) Specific proteins associated with each pathway identified in (b). d,e) Heatmap depicting proteins involved in the IFN response and cell cycle pathways. f) GSEA revealing enrichment in the Ben‐Porath MYC MAX target pathway, p.adjust<0.05. g,h) Detection of (g) proteins related to the IFN response pathway and (h) CD19 and PI3K/AKT/c‐MYC on the surface of SEM cells treated with EVs for 4 or 24 h. i) Apoptosis in SEM cells treated for 24 h with fludarabine (1 µm), IFN‐γ (5 ng), and engineered EVs. PBS was used as a control. j) Apoptosis in SEM cells treated for 24 h with SC‐79 (5 µm), GSK690693 (5 µm), and engineered EVs. PBS was used as a control. 293‐EV as a negative control. The representative result of three independent experiments is shown. Each data point represents the means ± SD (*n* = 3). Statistical analysis was performed using the Student's *t*‐test for the unpaired data. Statistical significance: *** *p *< 0.001.

To better understand the mechanisms underlying the different killing capabilities of the modified EVs at the protein‐level, we performed a proteomic analysis to investigate the impact of treatment with different EVs on SEM target cells. Differentially expressed proteins (DEPs) are visualized in a circular heatmap that shows overall deregulation of proteins in SEM cells treated with CD19‐63‐293‐EV and CD19‐81‐293‐EV compared to those treated with CD19‐293‐EV, with distinct clustering of experimental groups (**Figure** **5**a; Figure S26, Supporting Information). Gene Ontology (GO) pathway enrichment analyses indicated the presence of downregulated proteins across specific subgroups (Figure 5b). The proteins associated with these pathways are illustrated in Figure 5c. Two heatmaps provide further details on the proteins involved in cellular responses to IFN response‐ and cell cycle‐related pathways. In SEM cells treated with CD19‐63‐293‐EV and CD19‐81‐293‐EV, the abundance of proteins associated with the IFN response and cell cycle pathways was significantly reduced compared to those treated with CD19‐293‐EV (Figure 5d,e; Figures S27 and S28, Supporting Information). The downregulation of cell cycle‐related proteins is consistent with the finding that optimized EVs possess better cytotoxicity against target cells, and the downregulation of IFN response‐related proteins is in line with the finding that inhibition of the IFN response enhances the ability of engineered EVs to kill B‐ALL target cells (Figure 1n). Furthermore, Gene Set Enrichment Analysis (GSEA) identified the Ben‐Porath MYC MAX target pathway as one of the most significantly enriched pathways (Figure 5f; Figure S29, Supporting Information). This suggests that the enhanced cytotoxicity of the optimized EVs is associated with a higher degree of disruption of the CD19/AKT/MYC axis, demonstrating that disruption of the CD19/AKT/MYC axis plays an important role in CD19‐targeting EV‐mediated target cell killing (Figure S4, Supporting Information).

To validate the results from the proteomic analysis, we measured the protein abundance of several key factors involved in the IFN response and cell cycle‐related pathways. Western blot analyses revealed that IRF7 and p‐STAT1 levels in target cells were significantly elevated following treatment with all CD19‐targeting EVs, indicating that the IFN response was invariably activated. However, compared to those treated with the unoptimized CD19‐293‐EVs, the levels of IRF7 and p‐STAT1 were markedly reduced in target cells treated with the optimized EVs, especially CD19‐81‐293‐EVs (Figure 5g; Figures S30–S32, Supporting Information). Also, we observed that CD19‐81‐293‐EV significantly reduced the RNA levels of *IRF7* in SEM cells compared to CD19‐293‐EV and CD19‐63‐293‐EV (Figure S33, Supporting Information). This result demonstrates that EV optimization suppresses the active IFN response, thereby enhancing EV‐mediated killing. Moreover, CD19, p‐AKT, and MYC levels in the target cells were significantly reduced following treatment with CD19‐targeting EVs, especially the optimized EVs (Figure 5h; Figures S34–S37, Supporting Information), suggesting that EV‐mediated cytotoxicity is tightly correlated with the degree of IFN response suppression and CD19/AKT/MYC axis disruption.

To investigate how the IFN response affects EV‐mediated target cell killing, we treated SEM cells with engineered EVs in the presence of either fludarabine, an IFN response inhibitor, or IFN‐γ, an IFN response activator. Treatment with fludarabine enhanced EV‐mediated target cell killing, CD19 depletion, and EV uptake, while treatment with IFN‐γ reversed these effects (Figure 5i; Figure S38a,b, Supporting Information). These results suggest that inhibition of the IFN response promotes EV uptake and CD19 endocytosis, thereby enhancing EV‐mediated target cell killing. Notably, while the optimized EVs resulted in near‐complete depletion of CD19, treatment with fludarabine still promoted EV‐mediated target cell killing, suggesting that a mechanism independent of the CD19/AKT/MYC axis disruption likely contributes to the regulation of EV‐mediated target cell killing via IFN response.

The ability of EVs to encapsulate damage‐associated molecular patterns (DAMPs) such as DNA, as well as their induction of an elaborate IFN response, led us to reason that the relatively low‐level of IFN response in target cells treated with the optimized EVs is due to extensive degradation of these EVs by lysosomes. To test this hypothesis, we stained the DNA contained in the EVs with Hoechst dye and compared the DNA contents of different engineered EVs within SEM cells (Figure S39a, Supporting Information). CD81‐dynabeads‐coupled flow cytometry was used to normalize the DNA contents of the modified EVs (Figure S39b, Supporting Information). As expected, cells treated with CD19‐81‐293‐EV, which exhibits the highest killing ability, had the lowest DNA content (Figure S39c–f, Supporting Information). Furthermore, as indicated by detection of p‐STAT1 and IRF7, CQ treatment of target cells abrogated the ability of optimized EV to alleviate the IFN response (Figure S40, Supporting Information). Taken together, these results indicate that the relatively low‐level IFN response in target cells is due to extensive degradation of CD19/EV complexes in the lysosome.

We next addressed the impact of disrupting the CD19/AKT/MYC signaling pathway on EV‐mediated target cell killing capability. Treatment with the AKT inhibitor GSK6906935^[^ ^55^, ^56^ ^]^ enhanced EV‐mediated target cell killing, and treatment with the AKT activator SC‐79^[^ ^57^ ^]^ reversed this effect (Figure 5j). These results suggest that disruption of the CD19/AKT/MYC signaling pathway is crucial for effective EV‐mediated target cell killing. Notably, while SC‐79 is able to partially inhibit EV‐mediated target cell killing, it is not able to fully block it, suggesting that another mechanism contributes to the regulation of EV‐mediated target cell killing.

### 2.9 Targeting Module‐Optimized EVs, Especially CD19‐81‐293‐EXO, have Excellent In Vivo Efficacy

**Figure 6:**
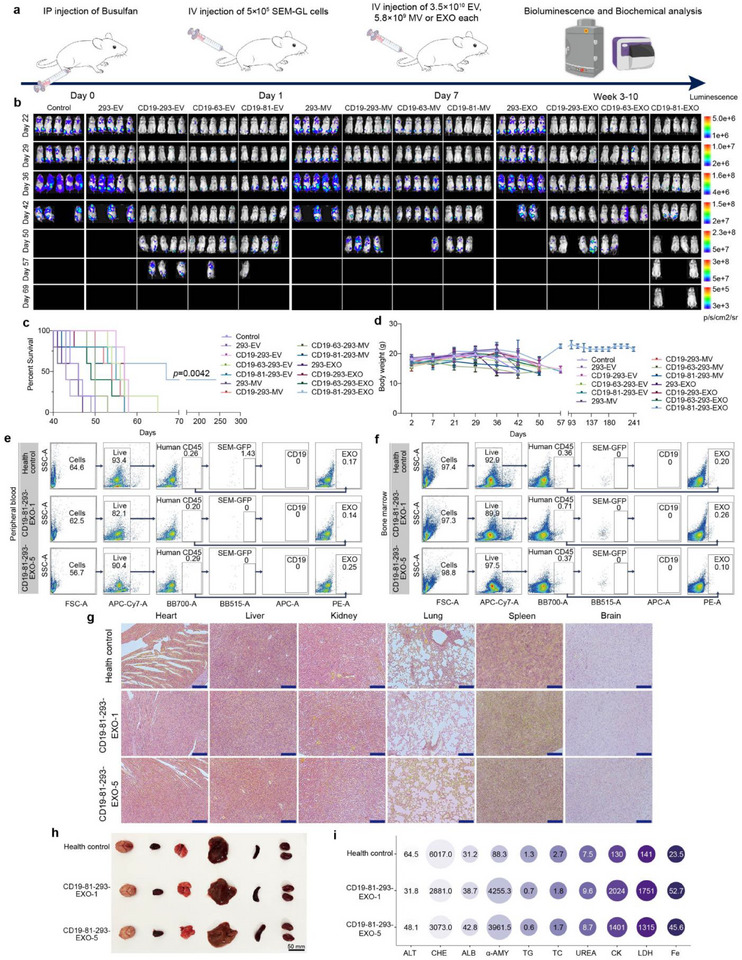
In vivo anti‐tumor capacity and biosafety evaluation. a) Administration schedule of CD19‐293‐EV/MV/EXO, CD19‐63‐293‐EV/MV/EXO, and CD19‐81‐293‐EV/MV/EXO treatments. b) NOD/SCID mice (*n *= 5) were transplanted with SEM‐Luc‐GFP cells and treated with engineered EVs. Mice underwent imaging on the indicated days after xenografting to assess leukemia progression. c) Percent survival of (b). CD19‐81‐293‐EXO‐treated group showed prolonged survival compared to the control (*p*=0.0042, log‐rank Mantel‐Cox test). d) Body weight of mice described in (b). Each data point represents the mean ± SD (*n* = 5). 293‐EV as a negative control. e,f) Number of human CD45‐labeled cells in the (e) peripheral blood and (f) bone marrow of healthy mice, CD19‐81‐293‐EXO‐1 mice, and CD19‐81‐293‐EXO‐5 mice, as determined by flow cytometry, with the percentage of SEM‐Luc‐GFP cells, CD19 protein levels, and total EXO‐mCherry expression. g) HE staining of organs 241 days post‐injection with CD19‐81‐293‐EXO. Scale bar: 200 µm. h) Gross morphology of organs 241 days post‐injection with CD19‐81‐293‐EXO. Scale bar: 50 mm. i) Serum biochemical analyses in healthy mice, CD19‐81‐293‐EXO‐1 mice, and CD19‐81‐293‐EXO‐5 mice. Each data point represents the mean ± SD (*n* = 3). PBS was used as a control. Scale bar: 200 µm.

To evaluate the in vivo therapeutic efficacy of the engineered EVs, NOD/SCID mice were transplanted with SEM‐Luc‐GFP cells. Successfully xenografted animals were randomly divided into groups and treated with various engineered EVs derived from the aforementioned cells at designated intervals. Since different EV isolation methods may retain different abundance of large proteins, which may be counted as EVs and affect accurate EV dosing,^[^ ^58^ ^]^ we therefore adjusted the dose (particle numbers) of EVs according to the expression levels of CD81 to ensure more accurate EV dosing. Bioluminescence imaging was used to confirm successful xenografting and monitor the progression of leukemia (**Figure** **6**a; Figure S41, Supporting Information). In line with the in vitro results, mice that received either PBS or high‐dose 293‐EV/MV/EXO treatment experienced rapid expansion of leukemia cells and an increase in mortality around day 48 post‐injection (Figure 6b). By comparison, the high‐dose engineered EVs exhibited enhanced anti‐tumor effects. These results reaffirm that while EVs derived from unmodified non‐killer cells lack the intrinsic capability to target and eliminate tumors, loading them with targeting modules confers target cell killing capability.

Interestingly, CD19‐81‐293‐EXO demonstrates excellent efficacy in eliminating SEM‐Luc‐GFP cells in vivo, as evidenced by the fact that two mice remained alive until the study was terminated on day 241 post‐injection (Figure 6c). During the course of CD19‐81‐293‐EXO treatment, two mice lost fluorescence signal from SEM‐Luc‐GFP, with no detectable signal since day 29 (Figure S42a, Supporting Information). This demonstrates that CD19‐81‐293‐EXO is highly effective at eradicating malignant B‐ALL cells, indicating its potential as a novel cell therapy strategy for treating B‐ALL.

**Figure 7:**
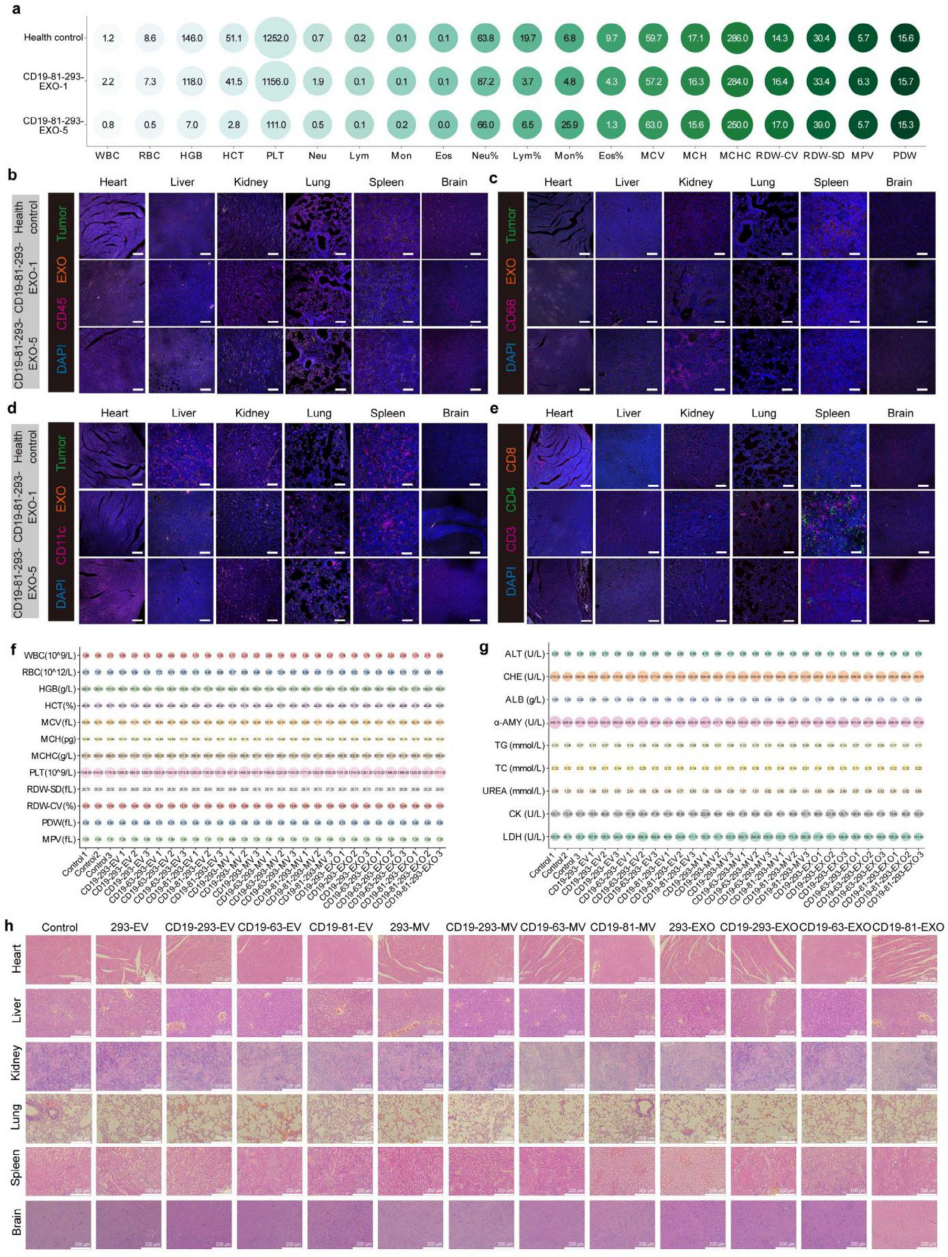
CD19‐81‐293‐EXO cleared SEM cells in vivo without residual EXOs and inflammatory response. a) Routine blood analyses in healthy mice, CD19‐81‐293‐EXO‐1 mice, and CD19‐81‐293‐EXO‐5 mice. b–e) Immunofluorescent staining of (b) CD45^+^ leukocytes; (c) CD68^+^ macrophages and monocytes; (d) CD11c^+^ dendritic cells; (e) CD3^+^, CD4^+^, and CD8^+^ T cells, SEM‐Luc‐GFP tumor cells, and EXOs in the organs of healthy control mice, CD19‐81‐293‐EXO‐1 mice, and CD19‐81‐293‐EXO‐5 mice. Scale bar: 100 µm. f–h) Routine blood tests (f), serum indicator levels (g), and HE staining (h) of organs 30 days post‐injection with engineered EVs. PBS was used as a control. 293‐EV as a negative control.

The CD19‐81‐293‐EXO‐1 and CD19‐81‐293‐EXO‐5 mice exhibited no signs of disease progression (e.g., weight loss or anorexia) throughout the 241 days of treatment (Figure 6d). To evaluate relapse potential and EXO persistence in vivo, we collected peripheral blood and bone marrow samples from sacrificed mice on day 241 and performed flow cytometry analysis. Notably, there were no detectable leukemic cells or EXOs in the peripheral blood and bone marrow of the CD19‐81‐293‐EXO‐1 and CD19‐81‐293‐EXO‐5 mice (Figure 6e,f). Although these mice exhibited leukemia‐associated symptoms, such as back flexion and joint swelling (Figure S42b, Supporting Information), treatment with CD19‐81‐293‐EXO did not result in serious side effects. Hematoxylin‐eosin (HE) staining showed that there was no significant cell necrosis or tissue damage in the organs of the mice treated with CD19‐81‐293‐EXO‐1 and CD19‐81‐293‐EXO‐5 on day 241 post‐injection (Figure 6g,h), though serum biochemical analyses revealed slight abnormalities in these mice (Figure 6i). CD19‐81‐293‐EXO‐5 mice also presented with mild anemia (**Figure** **7**a; Figure S43, Supporting Information). However, there were no signs of abnormal organ damage, indicating that treatment with EXOs did not adversely affect overall health (Figure 6h).

To investigate the immune system and inflammatory response in mice, immunofluorescence staining was performed. The results showed that CD19‐81‐293‐EXO treatment recruited similar levels of CD45+ leukocytes (Figure 7b); CD68^+^ macrophages and monocytes (Figure 7c); CD11c^+^ dendritic cells (Figure 7d); and CD3^+^, CD4^+^, and CD8^+^ T cells (Figure 7e) into the tumor compared to the control group. This suggests that treatment with CD19‐81‐293‐EXO does not induce a significant inflammatory response or promote immune cell aggregation.

### 2.10 Engineered EVs Demonstrate High Levels of Biosafety

To assess the biosafety of the engineered vesicles, NOD/SCID mice were administered these vesicles via tail vein injection. Their health was monitored over 30 days by evaluating their body weights, serum indices, and routine blood parameters. On day 30 post‐injection, there were no significant differences in body weights, serum indicator levels, or routine blood tests between the groups treated with engineered vesicles and the control group (Figure 7f,g; Figure S44, Supporting Information). Additionally, histological examination using HE staining showed no evidence of cell necrosis or tissue damage in the organs of the treated mice compared to the control group (Figure 7h). This indicates that the treatment with engineered EVs is safe and effective. The mild anemia and joint swelling observed in the CD19‐81‐293‐EXO‐1 and CD19‐81‐293‐EXO‐5 mice are attributed to the onset of the disease. In conclusion, all the engineered EVs demonstrate a favorable biosafety profile, supporting their potential application in anti‐tumor therapies without adverse effects on normal tissues.

## 3 Discussion

Current FDA‐approved CAR T‐cell therapies mainly use highly personalized CAR‐T cell products from patients’ T cells, requiring production techniques that are complicated, time‐consuming, and highly expensive. The manufacturing procedure may delay treatment, and not all patients can successfully produce CAR‐T cell products. Allogeneic off‐the‐shelf CAR T‐cell therapy could be a solution that can circumvent both the complexity and the costs associated with autologous CAR‐Ts. However, these CAR‐T products usually require additional genetic engineering to minimize the risk of CAR T‐cell rejection and graft‐versus‐host disease (GVHD). It is therefore imperative that efforts are made to establish CAR‐T‐derived EVs as an alternative universal therapeutic approach to CAR‐T therapy.^[^ ^59^ ^]^

**Figure 8:**
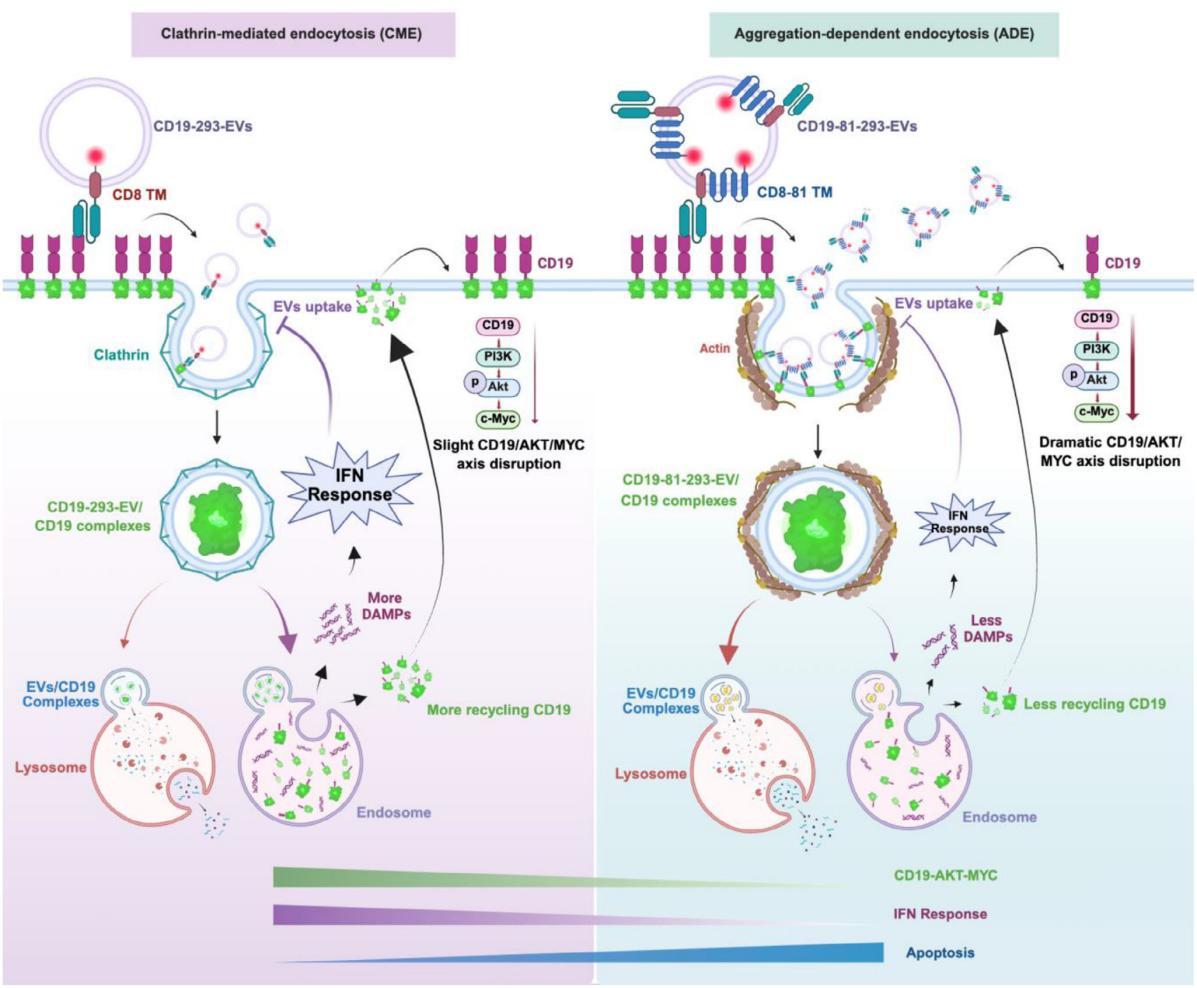
Schematic representation of a cell therapy strategy in which bioengineered EVs enhance anti‐tumor capacity through inhibition of the IFN response to enhance the targeted delivery of EVs and promote the endocytosis of CD19 antigens.

EVs possess inherent advantages, including high stability, the ability to penetrate tissue cells, and good biocompatibility. These properties make them potentially effective tools in cancer immunotherapy. Since unmodified non‐killer cell‐derived EVs lack the intrinsic capability to target and eliminate tumors, several studies have focused on EVs derived from killer cells, especially CAR‐T and CAR‐NK cells.^[^ ^59^ ^]^ This study provides proof‐of‐concept that non‐killer cell‐derived EVs can acquire the capability to kill target cells through the loading of targeting modules, providing a foundation on which innovative techniques to manufacture therapeutic engineered EVs can be developed. Our strategy suggests that therapeutic engineered EVs can be obtained from easy‐to‐culture non‐killer cells, eliminating the need for sophisticated surface modification and drug loading, which is often complex, inefficient, and detrimental to the structure of the EVs. Moreover, EV‐mediated target cell killing can be enhanced through structural optimization of the targeting module using a chimeric CD8‐CD63 or CD8‐CD81 transmembrane domain. The findings of this study provide fresh insight into new approaches for generating therapeutic engineered EVs and offer a solid model on which the development of “off‐the‐shelf” cancer therapies can be based (**Figure** **8**).

Many endocytosis routes have been proposed to be involved in EV uptake.^[^ ^60^ ^]^ However, it is unclear which factors determine the endocytic pathway taken or how these different pathways impact EV‐mediated target cell killing. In this study, we showed that the abundance of targeting modules on the EV surface contributes to the endocytosis pathway undertaken. While EVs with a moderate abundance of targeting modules prefer the CME pathway, EVs with a significant abundance of targeting modules prefer the ADE pathway. The ADE pathway is associated with enhanced EV uptake and EV‐mediated target cell killing. Together, the findings of this study demonstrate that EV‐mediated target cell killing can be modulated by switching the endocytosis route, providing a novel method by which to enhance EV function.

Natural EVs are known to have a low rate of absorption by their recipient cells.^[^ ^60^ ^]^ This study suggests a possible explanation for this: EV‐mediated activation of the IFN response in recipient cells. Similar phenomena have been observed in viral infections, wherein the virus triggers the IFN response in host cells which, in turn, inhibits viral endocytosis.^[^ ^61^ ^]^ In this study, we establish a tight correlation between diminished IFN response and enhanced EV uptake EV‐mediated killing of target cells. This relationship suggests that modulation of the IFN response, which is easily achieved by clinically available drugs, promotes the therapeutic efficacy of EVs by improving the rate of EV uptake.

Through investigation of the mechanisms by which target cells are susceptible or resistant to EV‐mediated killing, we established that target cells rely on target antigens for survival, a determining factor crucial for the successful application of engineered EVs in cancer therapies. In addition to CD19, many of the commonly used CAR‐T target antigens are key to the survival of the tumor cells. For example, BCMA is essential for the survival of multiple myeloma cells,^[^ ^62^ ^]^ and CD22‐deficient B cells exhibit reduced proliferation and enhanced turnover rates.^[^ ^63^ ^]^ This property makes us confident that, while our prototype design is currently only tested on targeting CD19 in B‐ALL, our technology and strategies can be easily adapted to target many other types of tumors by substituting the CD19‐scFv with another antigen‐targeting module. In addition, the high uptake rate of these optimized EVs, especially CD19‐81‐293‐EXOs, makes them excellent drug delivery vehicles. For instance, these EVs could be loaded with reactive oxygen species (ROS) to disrupt the tumor microenvironment, inhibit tumor blood vessel formation, and induce tumor cell death.^[^ ^64^ ^]^ Alternatively, they could carry therapeutic miRNAs or potent oligonucleotides, expanding their application to various diseases, or even be used to boost the efficacy of existing CAR‐T therapies.

## 4 Experimental Section

### Cell Lines and Primary Cells

SEM (RRID: CVCL_0095), REH (RRID: CVCL_1650), Kasumi (RRID: CVCL_0589), THP1 (RRID: CVCL_0006), and Raji (RRID: CVCL_0511) cell lines were obtained from DSMZ (2018). KOPN8 (RRID: CVCL_1866), HEK‐293 (293, RRID: CVCL_0063), and NALM6 (RRID: CVCL_0092) cell lines were obtained from the American Type Culture Collection (ATCC, 2016). All cell lines were routinely tested for mycoplasma using a Mycoplasma Stain Assay Kit (C0296, Beyotime) and authenticated by Short Tandem Repeat (STR) profiling. Peripheral blood mononuclear cells (PBMCs) were isolated from healthy volunteer donors using a human peripheral blood lymphocyte separation solution (TBDscience). CD3+ T cells were isolated using EasySep Human T Cell Isolation Kit (STEMCELL Technologies) and then cultured in CTS T Cell Expansion medium (Thermo) containing 10% fetal bovine serum (FBS), 100 IU mL^−1^ human IL‐2, 50 IU mL^−1^ human IL‐7, 50 IU mL^−1^ human IL‐15, and 50 IU mL^−1^ IL‐21 (Peprotech) and stimulated with Dynabeads CD3/CD28 (Invitrogen). Cells were grown at 37 °C in 5% CO_2_ in RPMI 1640 (L210KJ, BasalMedia) and Dulbecco's modified Eagle's medium (DMEM, L110KJ, BasalMedia) supplemented with 10% fetal FBS and 1% penicillin and streptomycin.

### Plasmid Constructions

Fragments encoding CD19 anergic CARs (scFv), CD8 Hinge, CD28/4‐1BB co‐stimulatory, and CD3 ζ‐chain signaling domains were inserted into the lentiviral vector pCDH‐T2A‐copGFP (System Biosciences). Fragments encoding CD19 anergic CARs (scFv) that lack co‐stimulatory and ζ‐chain signaling domains were inserted into the lentiviral vector pHR (addgene). The transmembrane domain were CD8, CD63, and CD81. The mCherry sequence was inserted at the C‐terminus of the transmembrane domain.

### Preparation and Purification of EVs, MVs, and EXOs

Cell culture medium was sequentially centrifuged at 300 × g for 10 min and then at 2000 × g for 10 min. The resultant supernatant was then centrifuged at 15000 rpm for 30 min. MVs were obtained by discarding the supernatant and resuspending the precipitate in Dulbecco's phosphate‐buffered saline (DPBS, BasalMedia). The resuspension was filtered through a 0.22 µm filter (Millex) and subjected to ultracentrifugation (Beckman optima L‐80XP) at 30000 rpm for 2 h to pellet EXOs. The EXO pellets were dissolved and resuspended in PBS. The resultant filtered supernatant was added to a Millipore 100 kDa ultrafiltration tube (UFC910008, Merck) and centrifuged at 5000 rpm for 1 h to yield EVs. The entire collection process was maintained at 4 °C. For serum EXO isolation, serum was ultracentrifuged at 30000 rpm for 2 h to pellet the EXOs and the supernatant was transferred into a fresh tube. Nanoparticle tracking analysis was used to quantify the total protein concentration of the EVs, MVs, and EXOs.

### Flow Cytometry

The CellTrace Violet Proliferation Kit (C34557, Invitrogen) was used for cell labeling. Human CD19‐APC (561 742) and CD45‐BB700 (746 090) antibodies were obtained from BD Biosciences. APC Anti‐DDDDK tag (ab72569) antibody was obtained from Abcam. Alexa Fluor 488 anti‐STAT1 Phospho (686 410), TruStain FcX PLUS anti‐mouse CD16/32 (S17011E), and APC anti‐IRF7 (656 012) antibodies were obtained from BioLegend. CD81‐FITC antibody (561 956) and human CD81 flow detection reagent (10622D) used in EXO labeling were obtained from Thermo. Apoptosis was measured using an Annexin V Apoptosis Detection Kit (556 547, BD Bioscience). IFN‐γ protein (WX328991) was obtained from ABclonal. Fludarabine (T1038) and CQ (54 057) were obtained from TargetMOI. Triciribine (S1117), GSK690693 (S1113), SC‐79 (S7863), and cytochalasin D (S8184) were obtained from Selleck. Flow cytometry was performed on an LSRFortessa (X20, BD Biosciences). Data were analyzed using the FlowJo software (10.8.1).

### Transmission Electron Microscope (TEM)

EVs, MVs, and EXOs were mounted on carbon adhesive tape and imaged on a scanning TEM as previously described.^[^ ^48^ ^]^ For negative staining TEM analysis, EVs, MVs, and EXOs were placed on a carbon‐coated grid and allowed to settle for 2 min. The samples were blotted and negatively stained with 10 µL 3% aqueous uranyl acetate for 2 min. Following the stain, the grid was blotted and air‐dried. Grids were imaged with a TEM (HT7700, Hitachi) operating at 100 kV.

### Nanoparticle Tracking Analysis

NTA was performed on a NanoSight NS300 (Malvern Panalytical) to measure the size distribution and concentration of EVs, MVs, and EXOs as previously described.^[^ ^50^ ^]^ The samples were diluted in 0.1 m PBS for 10^9^ and 10^10^ per mL particle count. The camera focus was adjusted to make the particles appear as sharp dots. Each test was repeated three times.

### Flow Cytometry Analysis of CD81‐Positive EXOs

Following resuspension by vortex for 30 s, 20 µL of bead solution per sample was transferred to a tube containing 1 mL of assay buffer consisting of 0.1% bovine serum albumin (BSA) in DPBS. The tube was then placed in a magnetic separator for 2 min to isolate the beads and allow for the removal of the supernatant. Following this, 1 mL of assay buffer and 10 µL of pre‐enriched EXO sample were added to the beads and incubated overnight at 4 °C with end‐over‐end mixing. The tube was placed in the magnetic separator for 2 min to allow for the removal of the supernatant, and 1 mL of assay buffer and 1 µL of CD81‐FITC antibody (561 956, Thermofisher) were added to the beads prior to incubation at 4 °C for 30 min on an orbital shaker (1000 rpm) in the dark. An additional 1 mL of assay buffer was added to each tube, and then, the tubes were placed in the magnetic separator for 2 min to remove the supernatant. This wash step was repeated once before a final 100 µL of assay buffer was added to each sample to perform flow cytometry analysis.

### Preparation and Purification of Hoechst‐Stained EVs

Hoechst‐stained EVs were obtained by adding 1 µL Hoechst 33 342 (62 248, Thermofisher) to 20 µL of EVs (3.5×10^9^ EVs) and incubating at 4 °C for 10 min. Stained EVs were added to a Millipore 100 kDa ultrafiltration tube (UFC910008, Merck) and centrifuged at 5000 rpm for 1 h.

### Immunoblots

Cells, EVs, MVs, and EXO pellets were lysed in erythrocyte blood cell (EBC) lysis buffer, and protein concentration was determined using a bicinchoninic acid (BCA) assay kit (P0009, Beyotime). Proteins were separated on 10% sodium dodecyl sulfate‐polyacrylamide gel electrophoresis (SDS‐PAGE) gels and transferred to a polyvinylidene difluoride (PVDF) membrane. The membrane was washed, blocked with 5% skim milk, and probed overnight with primary antibodies. Human anti‐CD63 (ab193349, 1/1000) and anti‐CD9 (ab92726, 1/1000) antibodies were obtained from ABclonal Technology. Anti‐CD81 (UR52343, 1/1000) antibodies were obtained from Umibio Science and Technology Group. Human anti‐CD19 (3574S, 1/1000), anti‐Akt (9272S, 1/1000), anti‐IRF7 (72073S, 1/1000), anti‐STAT1 (14994S, 1/1000), anti‐phosphor‐STAT1 (p‐STAT1, 9167S, 1/1000), anti‐phosphor‐Akt (anti‐p‐Akt, 4060S, 1/2000), and anti‐GRP78 (3177T, 1:1000) antibodies were purchased from Cell Signaling Biotechnology. Human anti‐MYC antibody (2278S, 1/1000) was obtained from Santa Cruz Technology. Human anti‐GAPDH antibody was obtained from Sigma Aldrich. Following overnight incubation, the membrane was incubated in goat anti‐rabbit IgG (7074S, Cell Signaling Biotechnology) or goat anti‐mouse IgG (7076S, Cell Signaling Biotechnology) secondary antibodies at room temperature for 1 h. An enhanced chemiluminescence (ECL) Western blot analysis system (Millipore) was used to visualize proteins on the membrane on an Amersham Imager 600 (General Electric Company), and the intensity of the bands was measured using ImageJ software. Each experiment was performed at least three times (at least three animals).

### Super‐Resolution Imaging

293‐CD19‐GFP cells expressing CD19‐GFP fusion labeled with EVs, MVs, and EXO pellets expressing mCherry were seeded in cell culture imaging dishes. The cells were stained with LysoBrite (22 642, AAT Bioquest) blue dye for 30 min and then washed. They were seeded in 20‐mm glass bottom dishes (D35‐20‐1.5‐N, Cellvis) overnight. Images were acquired on the Nikon spinning disc microscope (CSU‐W1) imaging system, and Fiji software was used to generate the images.

### Mouse Studies and In Vivo Imaging

SEM cells simultaneously expressing GFP and luciferase (SEM‐Luc‐GFP), as described previously, were used.^[^ ^65^ ^]^ NOD/SCID mice were purchased from GemPharmatech Company and used in all experiments (five mice in each test and control group). First, 5×10^5^ luciferase‐expressing cells were intravenously injected via the tail vein into NOD/SCID mice. The mice were then then administered 3.5×10^9^ or 3.5×10^10^ EVs, 1×10^9^ or 5.8×10^9^ MVs, 1×10^9^ or 5.8×10^9^ EXOs from 293, CD19‐293, CD19‐63‐293, and CD19‐81‐293 cells on a schedule beginning 7 days after xenograft. Total body bioluminescence was quantified at indicated time points. All animal studies were approved by the Charles River Committee.

### Hematoxylin and Eosin (HE) Staining

Histological analysis of the organs from the NOD/SCID mice was performed on day 30 or 241 post‐injection. Organs were fixed in 4% formalin and then dehydrated in gradient concentrations of ethanol (70–100%) and xylene. The samples were embedded in liquid paraffin, sliced, and imaged on an inverted microscope. PBS was used as a control.

### Blood Biochemical Analysis

Blood was collected from NOD/SCID mice on day 30 or 241 post‐injection in the presence of ethylenediaminetetraacetic acid (EDTA) anticoagulant and heparin sodium. Blood serum levels of albumin (ALB), alanine aminotransferase (ALT), total cholesterol (TC), triglycerides (TGs), cholinesterase (CHE), urea, creatine kinase (CK), lactate dehydrogenase (LDH), and alpha‐amylase (α‐AMY) were measured using an automatic biochemical analyzer (iCubio, China).

### Immunohistochemistry

Immunohistochemistry was performed on NOD/SCID mice organs on day 241 post‐injection of CD19‐81‐293 EXOs (5.8×10^9^ EXOs each). Organs were fixed in 4% paraformaldehyde, dehydrated in an automatic dehydrator TSJ‐II (Aihua, China), embedded in paraffin, and sectioned at 0.5‐mm thickness using a rotary microtome RM 2016 (Leica, Germany). To remove paraffin, tissue slices were sequentially washed in xylene (1330‐20‐7, Ling Feng, China) for 15 min and then in anhydrous ethanol (100 092 183, China National Pharmaceuticals), 85% alcohol, 75% alcohol, and distilled water for 5 min. The tissue sections were then placed in a repair cassette containing EDTA antigen repair buffer, pH 8.0 (Sangon Biotech, China) for antigen repair in a microwave oven (P70D20TL‐P4, GALANZ, China). Following this, the sections were dried using 3% BSA (A8010, solarbir, China) in a closed container for 30 min. Primary antibodies anti‐CD11C (97 585, CST), anti‐CD45 (60 287, Proteintech Group), anti‐CD68 (28 058, Proteintech Group), anti‐CD3 (ab16669, Abcam), anti‐CD4 (ab183685, Abcam), and anti‐CD8 (ab217344, Abcam) were added dropwise to the sections and incubated overnight at 4 °C. After washing thrice with PBS for 5 min each, the sections were incubated with the secondary antibody IgG (074‐1506, KPL) for 50 min at 37 °C and then with DAPI (ZLI‐9557, Zhongshan, China) for 10 min at 25 °C. Finally, the sections were incubated with an autofluorescence quencher for 5 min (E675011, Sangon Biotech, China), rinsed under running water for 10 min, and imaged using a fluorescence microscope FV4000 (OLYMPUS, Japan).

### RNA Sequencing and Protein Identification by Mass Spectrometry (MS)

Total RNA was extracted from cells using 1 mL TRIzol reagent (ThermoFisher) for every 1 × 10^6^ cells. Bulk RNA sequencing libraries were prepared using the Illumina TruSeq library construction kit and GISEQ‐500 PE100 (BGI Technology Service, Wuhan, China). Differentially expressed genes (DEGs) were identified using the R package “DESeq2” with raw read counts as input, based on the criteria of |log_2_FoldChange| > 1 and *p *< 0.05. Protein samples were sent to the Public Technology Platform of Shanghai Jiaotong University School of Medicine and analyzed using label‐free LC‐MS. Differentially expressed proteins (DEPs) were identified via the R package “limma” with log2‐transformed and normalized protein expression data as input, based on the criteria of |log_2_FoldChange| > 1 and *p *< 0.05.

### Functional Enrichment Analysis of Differentially Expressed Proteins (DEPs)

The functional characteristics of DEPs were identified via GO protein annotations. Pathway enrichment was carried out using the Kyoto Encyclopedia of Genes and Genomes (KEGG) database and GSEA. DEPs were identified by the limma package in R version 4.3.2. GO and KEGG enrichment analyses were conducted using the Database for Annotation, Visualization and Integrated Discovery (DAVID) platform, while GSEA pathway enrichment analysis was conducted using the clusterProfiler package in R version 4.3.2. Statistical significance was set at *p *< 0.05. All visualizations were generated with ggplot2 (v3.4.2).

### Reverse Transcription qPCR

SEM cells were seeded (1 × 10^6^ cells per well in 6‐well culture plate). 293‐EVs and engineered EVs were added to the cells in a final culture volume of 2 mL for co‐cultured. engineered EVs exposed cells were harvested at 24 h for cellular RNA extraction and cDNA preparation. Fold change was calculated by comparing control (293‐EVs) versus engineered EVs. Data was analyzed with RQ manager and expressed as fold change. GAPDH was utilized as a house‐keeping gene.

RNA was harvested with Super FastPure Cell RNA Isolation Kit (Vazyme, RC102‐01) as per manufacturer's protocol. Reverse transcription was performed with HiScript III 1st Strand cDNA Synthesis Kit (+gDNA wiper) (Vazyme, R312‐01) as per manufacturer's instruction. qPCR experiments were performed on an ABI 7500 thermal cycler (Thermo) in 20 µL reactions, with 10 nm of each primer and 2 µL of each cDNA preparation, using ChamQ SYBR qPCR Master Mix (Vazyme, Q341) as per manufacturer's instructions.

### Statistical Analysis

All statistical analyses were performed using GraphPad Prism 10 software. Student's *t*‐test was used to analyze the differences between groups.

### Ethics Approval and Consent to Participate

The animal study was performed in compliance with relevant regulatory standards. All animal studies were approved by Charles river committee (Approval No. P202410210001).

## Conflict of Interest

The authors declare no conflict of interest.

## Author Contributions

Z.H., C.L., Y.W., H.X., and Y.Z. contributed equally to this work. Z.H., C.L., and H.L. performed conceptualization, supervision, and project administration. Z.H., C.L., and Y.W. performed methodology. H.X. performed software. Z.H., Y.Z., and R.X. performed validation. Z.H. and M.W. performed formal analysis. Z.H., W.Z., and M.W. performed investigation. Z.H., Z.L.,Y.F., H.X., and H.H. provided resources. Z.H., R.P., and Y.Z. performed data curation. Z.H. performed visualization. Z.H., S.X., Y.W., and Y.F. performed draft preparation. Z.H., Y.Z., and H.H. performed wrote, ‐reviewed, and edited the draft. D.L. and H.L. performed funding acquisition.

## Supporting information



Supporting Information

## Data Availability

The data that support the findings of this study are available from the corresponding author upon reasonable request.
