# Supplementary material for: Enhancing Anti‐Tumor Effects of Engineered Extracellular Vesicles via Endocytosis Route Switching and Interferon Response Suppression
Source: Adv Sci (Weinh). 2025 Sep 23;12(46):e15472. doi: 10.1002/advs.202515472 (PMC12697815; doi:10.1002/advs.202515472)
Supplement: Supplementary file 1 — Supporting Information [file ADVS-12-e15472-s001.docx]

**Supporting information**

**Enhancing anti-tumor effects of engineered extracellular vesicles via endocytosis route switching and interferon response suppression**

Zixuan Huang^a*#^, Chaoqun Lu^a*^, Yixin Wang^a*^, Huajian Xian^a*^, Yuling Zheng^a*^, Ting Kang^b^, Rufang Xiang^c^, Shufeng Xie^a^, Minghui Wang^a^, Zeyi Li^a^, Xiaoli Xia^a^, Yaoyifu Yu^a^, Wenjie Zhang^d^, Huijian Zheng^d^, Renyao Pan^d^, Dan Li^a^, Chunjun Zhao^a#^, Han Liu^a#^.

^a^ Shanghai Institute of Hematology, State Key Laboratory of Medical Genomics, National Research Center for Translational Medicine at Shanghai, Ruijin Hospital, Shanghai Jiao Tong University School of Medicine and School of Life Sciences and Biotechnology, Shanghai 200025, China.

^b^ Department of Oncology, Xin Hua Hospital, Shanghai Jiao Tong University School of Medicine, Shanghai 200092, China.

^c^ Department of General Practice, Ruijin Hospital, Shanghai Jiao Tong University School of Medicine, Shanghai 200025, China.

^d^ Fujian Institute of Hematology, Fujian Provincial Key Laboratory on Hematology, Fujian Medical University Union Hospital, Fuzhou 350001, China.

* Contributed equally.

**^#^** Corresponding author.

**Han Liu** E-mail: liuhan68@sjtu.edu.cn

**Chunjun Zhao** E-mail: chunjun_zhao@163.com

**Zixuan Huang** E-mail: cherisehuang@sjtu.edu.cn

**Content**

Supplementary Fig. 1.

Supplementary Fig. 2.

Supplementary Fig. 3.

Supplementary Fig. 4.

Supplementary Fig. 5.

Supplementary Fig. 6.

Supplementary Fig. 7.

Supplementary Fig. 8.

Supplementary Fig. 9.

Supplementary Fig. 10.

Supplementary Fig. 11.

Supplementary Fig. 12.

Supplementary Fig. 13.

Supplementary Fig. 14.

Supplementary Fig. 15.

Supplementary Fig. 16.

Supplementary Fig. 17.

Supplementary Fig. 18.

Supplementary Fig. 19.

Supplementary Fig. 20.

Supplementary Fig. 21.

Supplementary Fig. 22.

Supplementary Fig. 23.

Supplementary Fig. 24.

Supplementary Fig. 25.

Supplementary Fig. 26.

Supplementary Fig. 27.

Supplementary Fig. 28.

Supplementary Fig. 29.

Supplementary Fig. 30.

Supplementary Fig. 31.

Supplementary Fig. 32.

Supplementary Fig. 33.

Supplementary Fig. 34.

Supplementary Fig. 35.

Supplementary Fig. 36.

Supplementary Fig. 37.

Supplementary Fig. 38.

Supplementary Fig. 39.

Supplementary Fig. 40.

Supplementary Fig. 41. Supplementary Fig. 42.

Supplementary Fig. 43.

Supplementary Fig. 44.

Table S1.


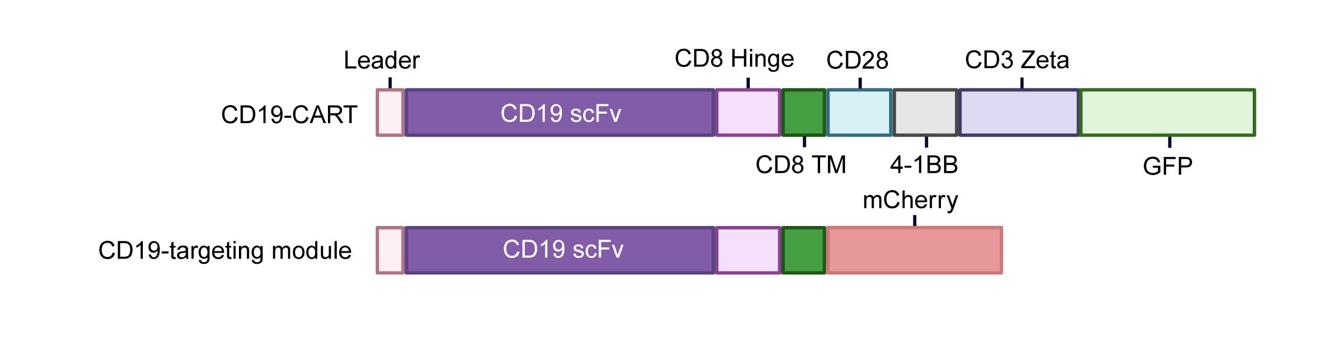


**Supplementary Fig. 1. Plasmid mapping of CD19-CART and CD19-targeting module.**


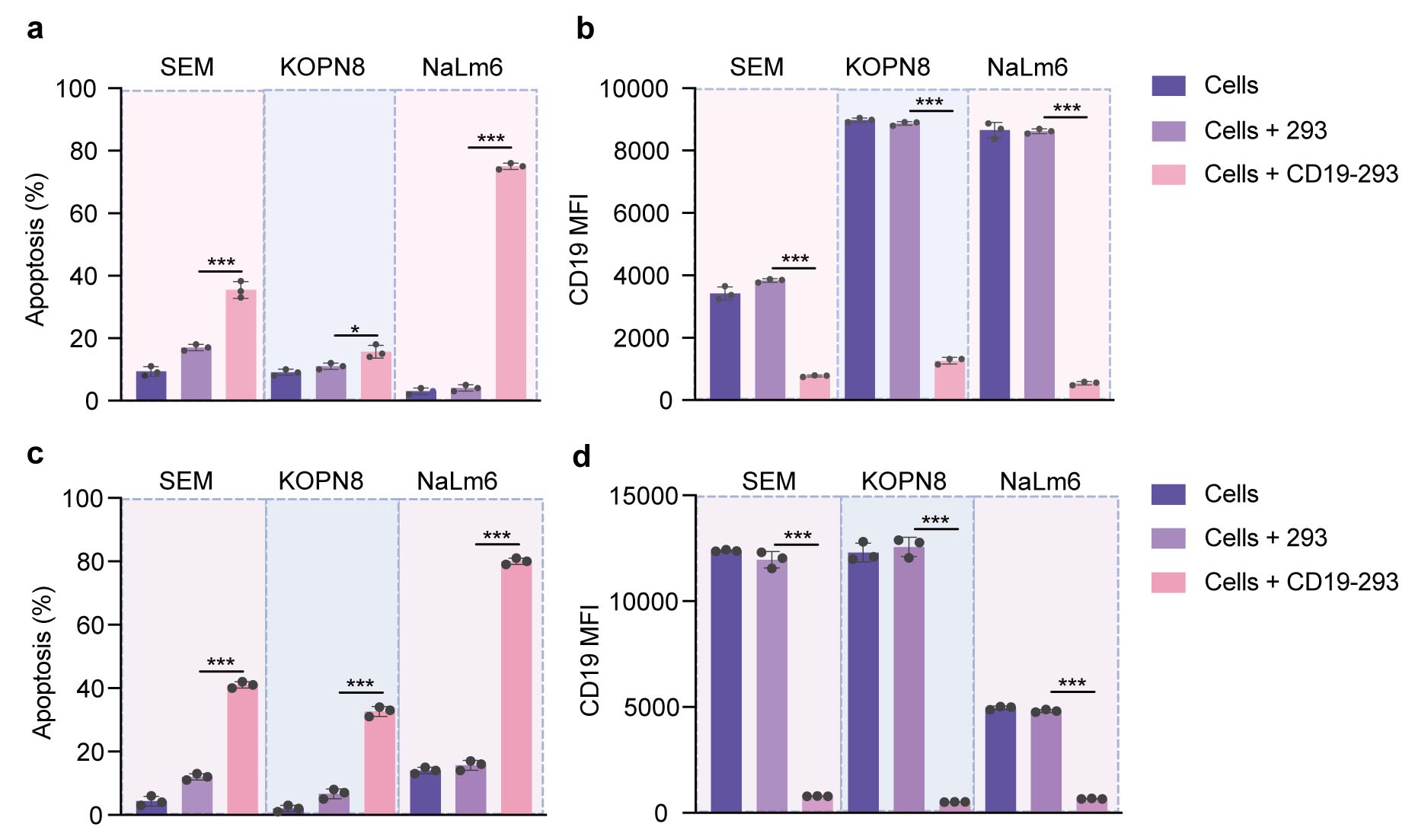


**Supplementary Fig. 2. Apoptosis and CD19 antigen expression in ALL cells co-cultured with CD19-293 cells.** SEM, KOPN8, and NaLm6 cells were co-cultured with 293 and CD19-293 cells for 24 **(a, b)** and 48 h **(c, d)** and then assessed by flow cytometry for target cell apoptosis **(a, c)** and MFI of CD19 **(b, d)**. The ratio of 293 or CD19-293 cells to SEM cells was 5:1. The representative result of three independent experiments is shown. Each data point represents the means ± SD (n=3). Statistical analysis was performed using Student’s *t*-test for the unpaired data. Statistical significance: *** *p*<0.001.


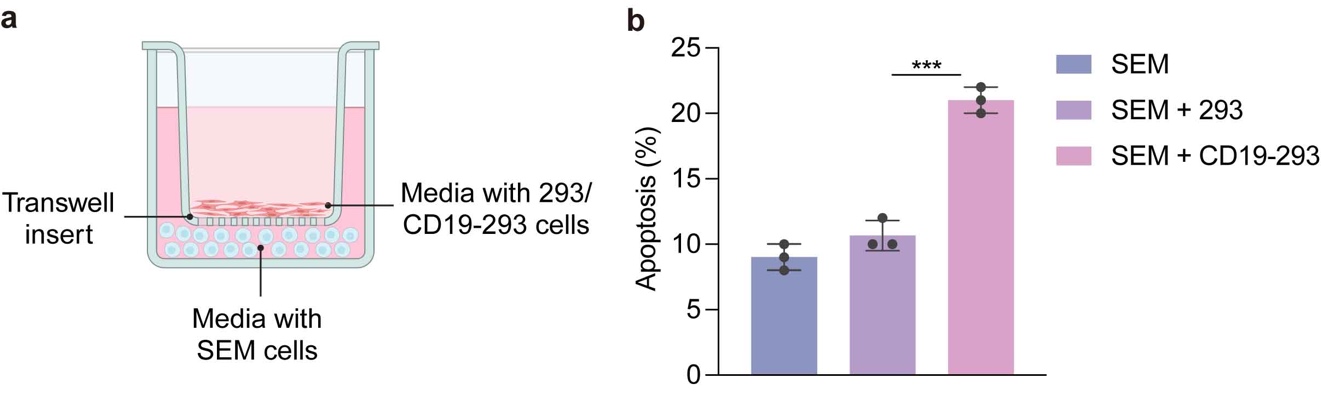


**Supplementary Fig. 3. Detection of the killing mechanism of CD19-293 cells. (a)** Schematic of the Transwell system used, wherein SEM cells were cultured in the bottom chamber and 293 cells or CD19-293 cells were cultured in the Transwell chamber. **(b)** After 48 h, SEM cell death was assessed via Annexin V staining. The ratio of 293 or CD19-293 cells to SEM cells was 2.5:1. The representative result of three independent experiments is shown. Each data point represents the means ± SD (n=3). Statistical analysis was performed using Student’s t-test for the unpaired data. Statistical significance: *** p<0.001. Image created with BioRender.com, used with permission.


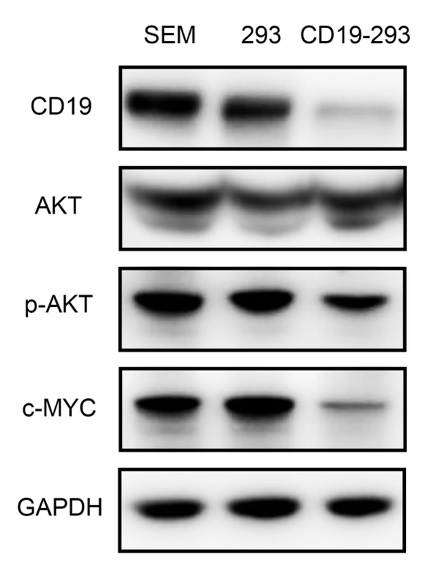


**Supplementary Fig. 4. Detection of SEM cell surface CD19 protein and PI3K/AKT/c-MYC protein expression.** Western blot detection of CD19, PI3K, AKT, and c-MYC expression in SEM cells following 24-h treatment with EVs.


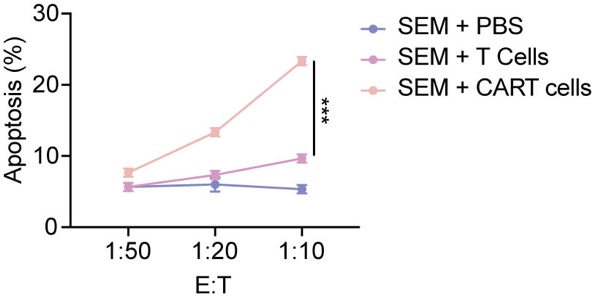


**Supplementary Fig. 5. Apoptosis of SEM cells cultured with CD19-CART cells.** SEM cells were co-cultured with T or CD19-CART cells for 24 h and then assessed by flow cytometry for target cell apoptosis. The ratio of effector cells to target cells (E:T) was 1:50, 1:20, or 1:10, as indicated. The representative result of three independent experiments is shown. Each data point represents the means ± SD (n=3). Statistical analysis was performed using Student’s *t*-test for the unpaired data. Statistical significance: *** *p*<0.001.


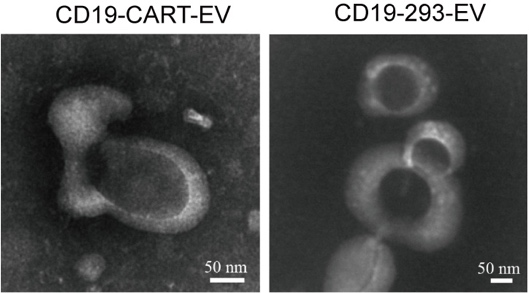


**Supplementary Fig. 6. Characterization of CD19-CART-EV and CD19-293-EV.** Transmission electron microscopy (TEM) was used to analyze the morphology of each sample group. Scale bar: 50 nm.


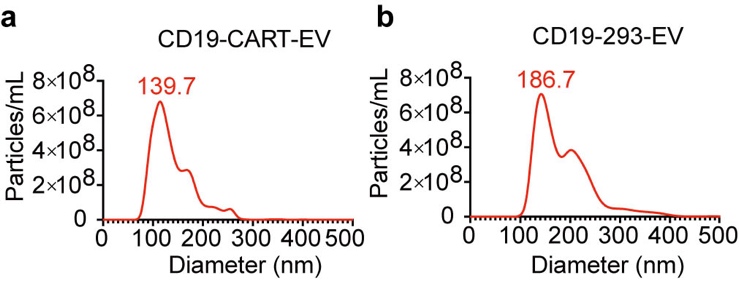


**Supplementary Fig. 7. Characterization of CD19-CART-EV and CD19-293-EV.** Nanoparticle tracking analysis (NTA) was used to determine **(a)** CD19-CART-EV and **(b)** CD19-293-EV particle sizes. Experiments were performed in biological triplicate


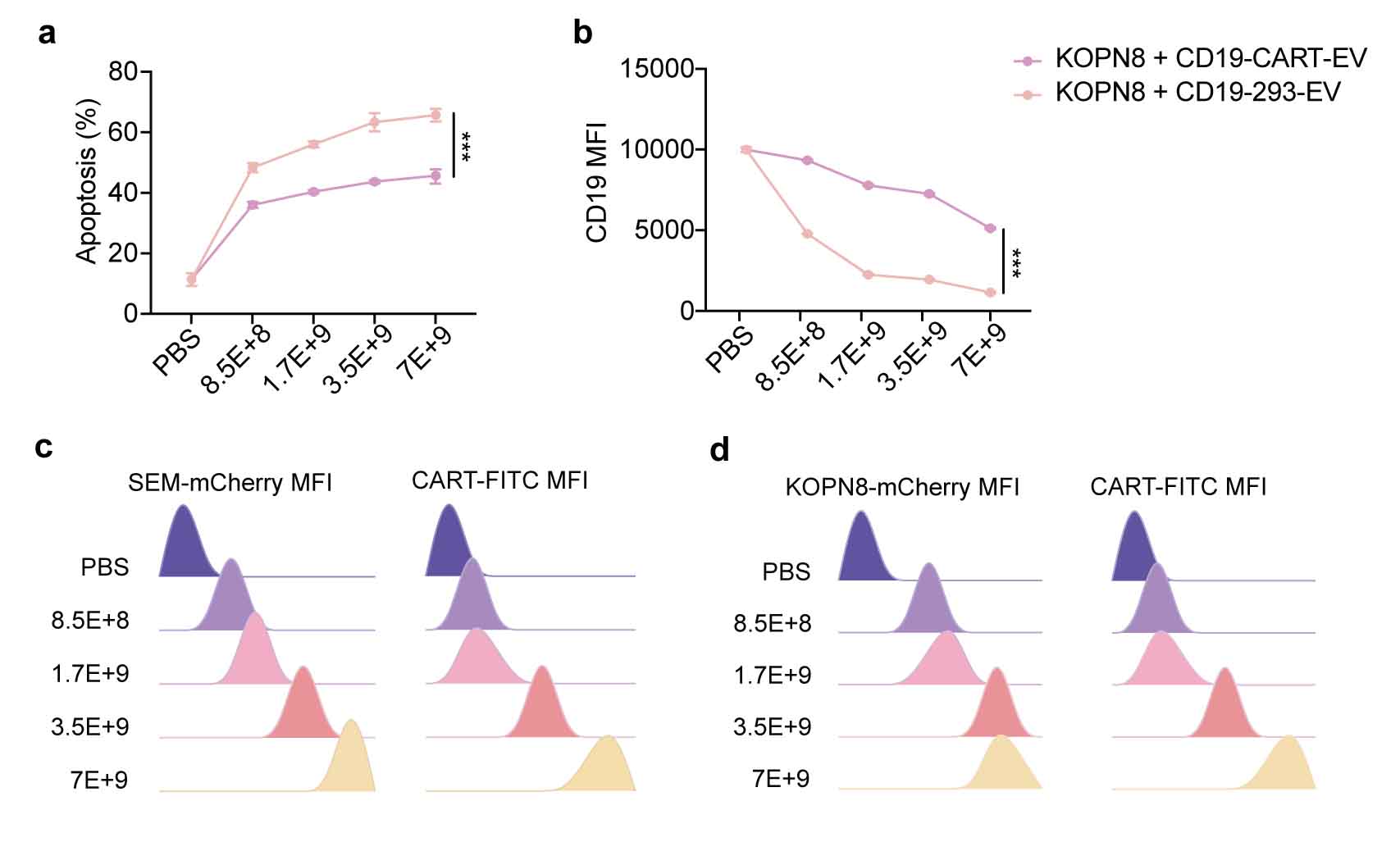


**Supplementary Fig. 8. Comparison of CD19-293-EV and CD19-CART-EV cytotoxicity.** SEM and KOPN8 cells were treated for 24 h with EVs of different particle numbers. **(a)** Target cell apoptosis, **(b)** MFI of CD19, and **(c, d)** EV uptake were detected by flow cytometry. The representative result of three independent experiments is shown. Each data point represents the means ± SD (n=3). Statistical analysis was performed using Student’s *t*-test for the unpaired data. Statistical significance: *** *p*<0.001.


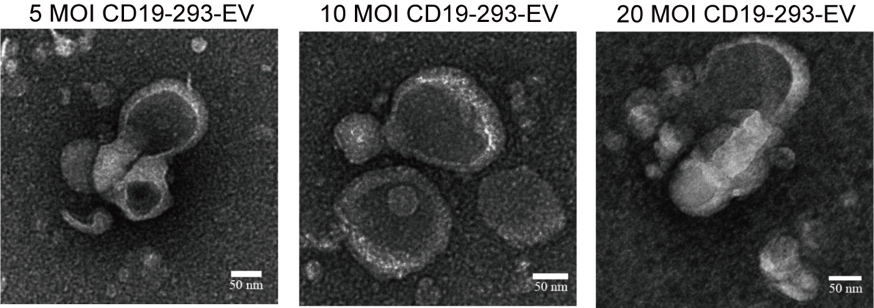


**Supplementary Fig. 9.** **Characterization of 5 MOI**, **10 MOI, and 20 MOI CD19-293-EV.** TEM characterization of EVs generated by 5, 10, and 20 MOI CD19-293 cells. Scale bar: 50 nm.


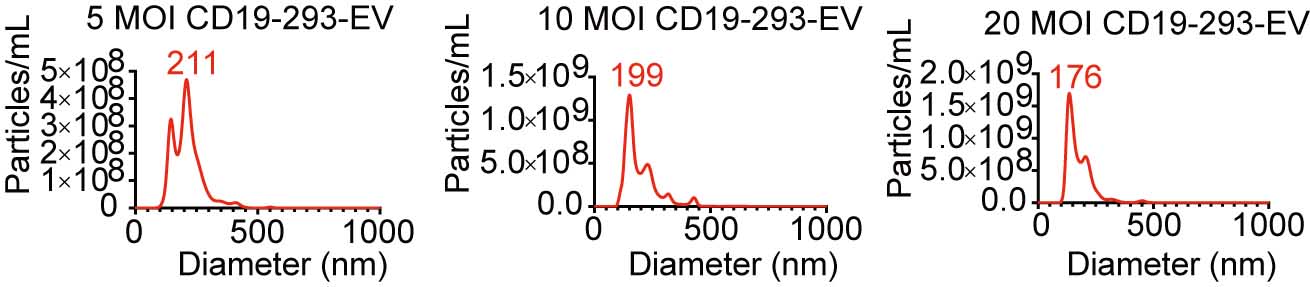


**Supplementary Fig. 10. Characterization of 5 MOI**, **10 MOI, and 20 MOI CD19-293-EV.** NTA characterization of EVs of 5, 10, and 20 MOI CD19-293 cells. Experiments were performed in biological triplicate.


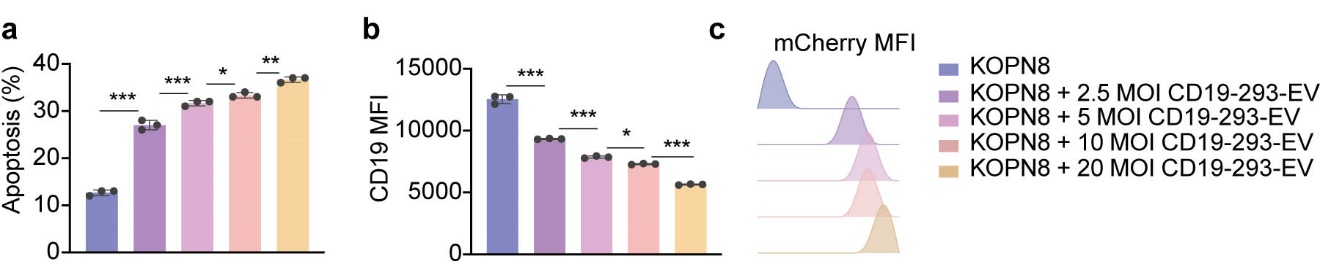


**Supplementary Fig. 11. Effect of EV treatment on apoptosis, CD19 MFI, and EV uptake in KOPN8 cells.** KOPN8 cells were treated for 24 h with EVs derived from different MOI CD19-293 and then assessed by flow cytometry for **(a)** apoptosis, **(b)** MFI of CD19, and **(c)** EV uptake. The representative result of three independent experiments is shown. Each data point represents the means ± SD (n=3). Statistical analysis was performed using Student’s *t*-test for the unpaired data. Statistical significance: *** *p*<0.001.


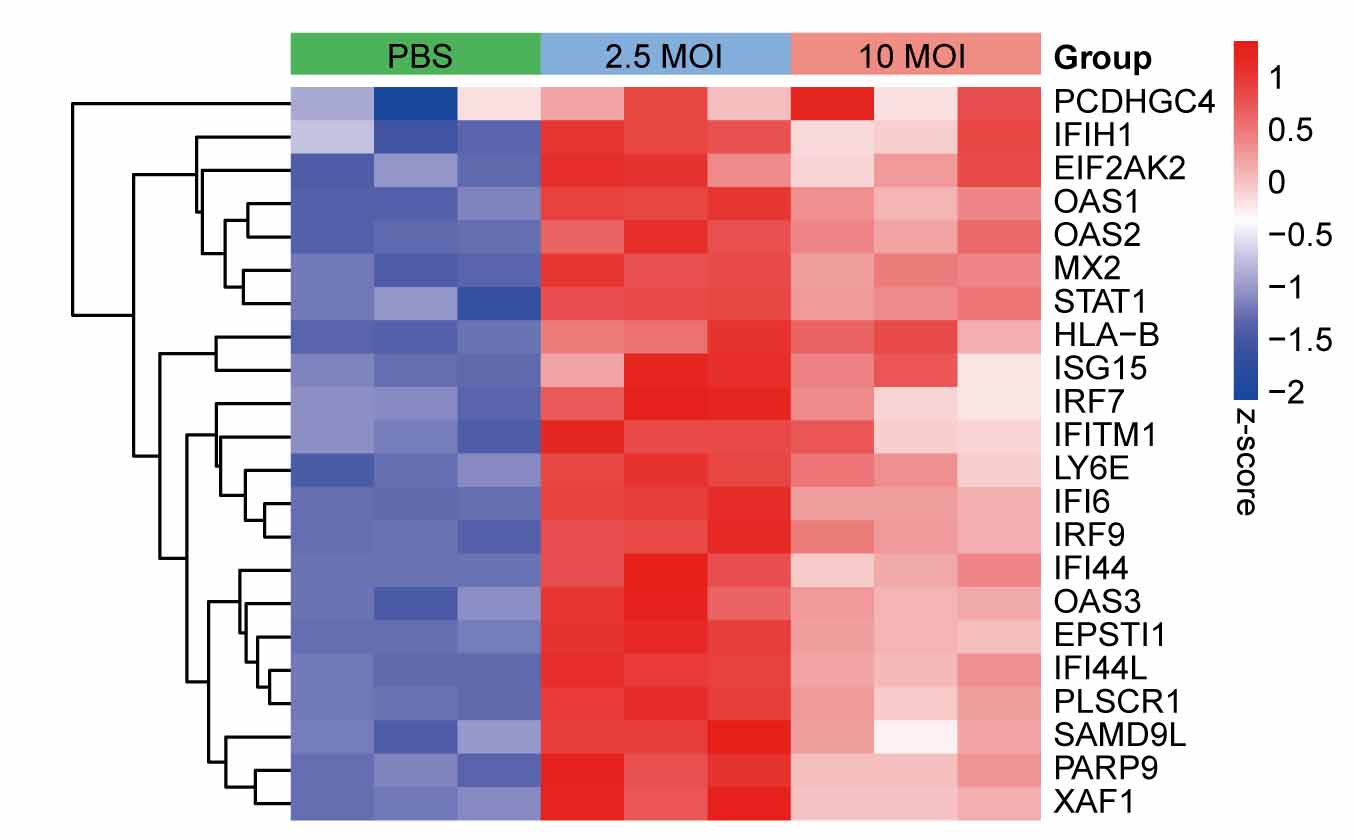


**Supplementary Fig. 12.** **Bulk RNA sequencing analysis of SEM cells after treatment with modified EVs.** RNA-seq analysis of SEM cells treated for 24 h with PBS, 2.5 MOI, or 10 MOI CD19-293-EV. The upregulated genes compared to the PBS group are shown in a clear heatmap, with significant differences (p<0.05).


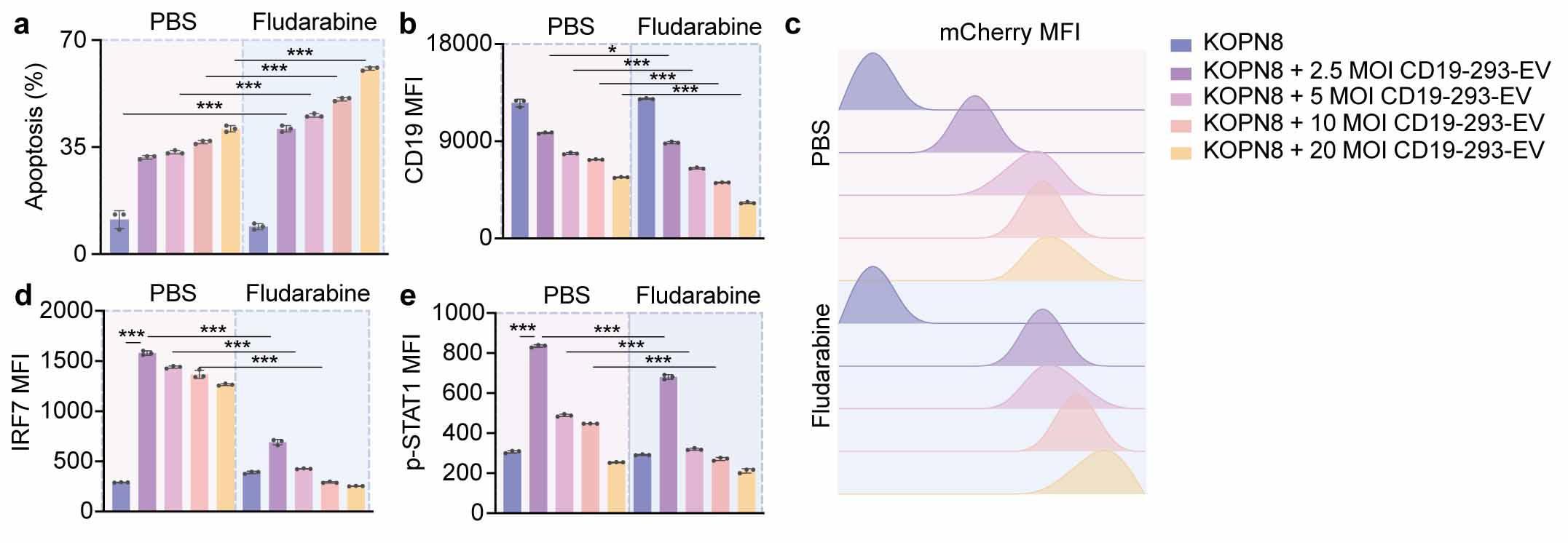


**Supplementary Fig. 13. Effect of fludarabine on apoptosis, CD19 MFI, and EV uptake in EV-treated KOPN8 cells.** KOPN8 cells were treated for 24 h with EVs of 1, 5, 10, and 20 MOI CD19-293 without or with fludarabine (1 μM) and then assessed by flow cytometry for **(a)** apoptosis, **(b)** MFI of CD19, **(c)** EV uptake, **(d)** IRF-7 MFI, and **(e)** p-STAT1 MFI. The representative result of three independent experiments is shown. Each data point represents the means ± SD (n=3). Statistical analysis was performed using Student’s *t*-test for the unpaired data. Statistical significance: *** *p*<0.001.


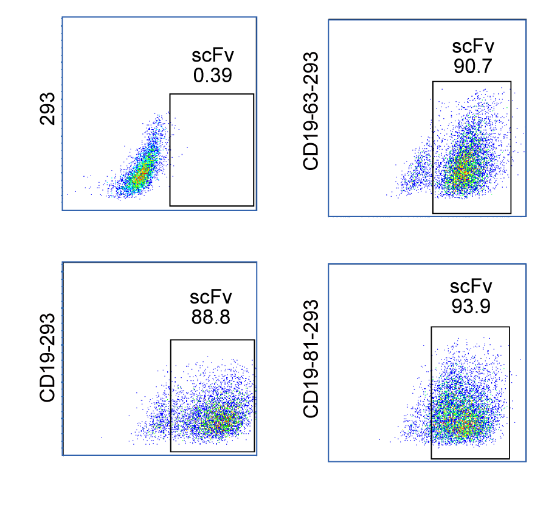


**Supplementary Fig. 14.** **Positive rates of target cells after lentiviral transfection.**


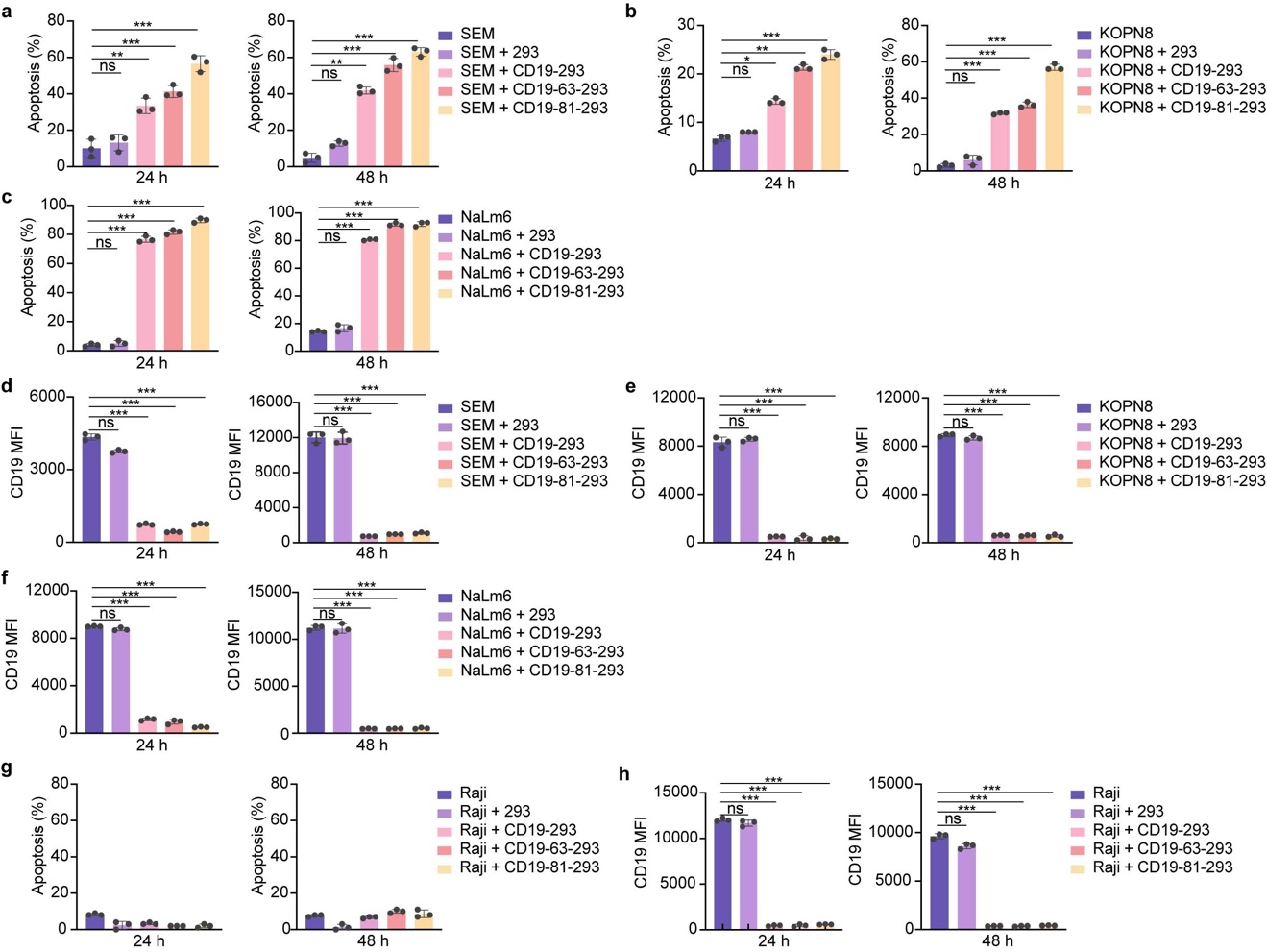


**Supplementary Fig. 15. Modified scFv cells co-cultured with B-ALL cells. (a-c)** Apoptosis and **(d**-**e)** target cell MFI of CD19 of SEM, KOPN8, and NaLm6 cells co-cultured with 293, CD19-293, CD19-63-293, and CD19-81-293 cells at 24 and 48 h. **(g)** Apoptosis and **(h)** CD19 MFI of Raji cells co-cultured with 293, CD19-293, CD19-63-293, and CD19-81-293 cells at 24 and 48 h. The ratio of effector cells to target cells (E:T) was 5:1. The representative result of three independent experiments is shown. Each data point represents the means ± SD (n=3). Statistical analysis was performed using Student’s *t*-test for the unpaired data. Statistical significance: *** *p*<0.001.


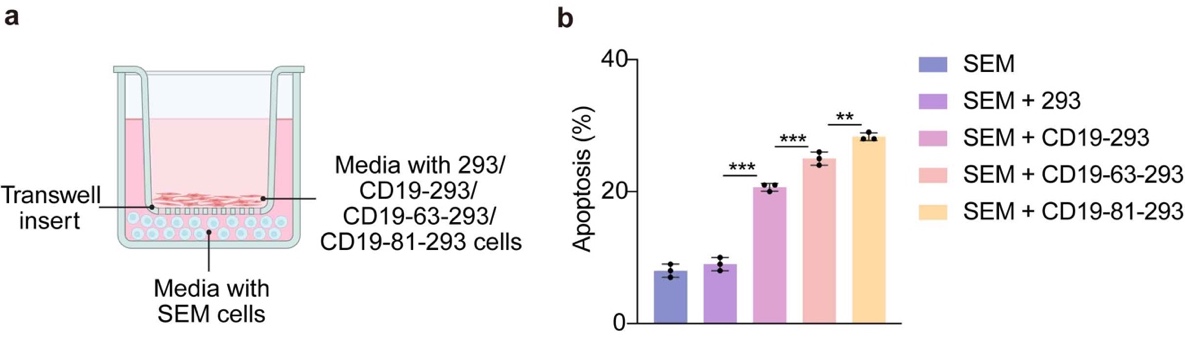


**Supplementary Fig. 16. Transwell co-culture of SEM and CD19-293/CD19-63-293/CD19-81-293 cells.** **(a)** Schematic of the Transwell system, wherein SEM cells were co-cultured in the bottom chamber and 293, CD19-293, CD19-63-293, or CD19-81-293 cells were cultured in the Transwell chamber. **(b)** After 48 h, SEM cell death was assessed via Annexin V staining. The ratio of 293, CD19-293, CD19-63-293, or CD19-81-293 cells to SEM cells was 5:1. The representative result of three independent experiments is shown. Each data point represents the means ± SD (n=3). Statistical analysis was performed using Student’s *t*-test for the unpaired data. Statistical significance: *** *p*<0.001. Image created with BioRender.com, used with permission.


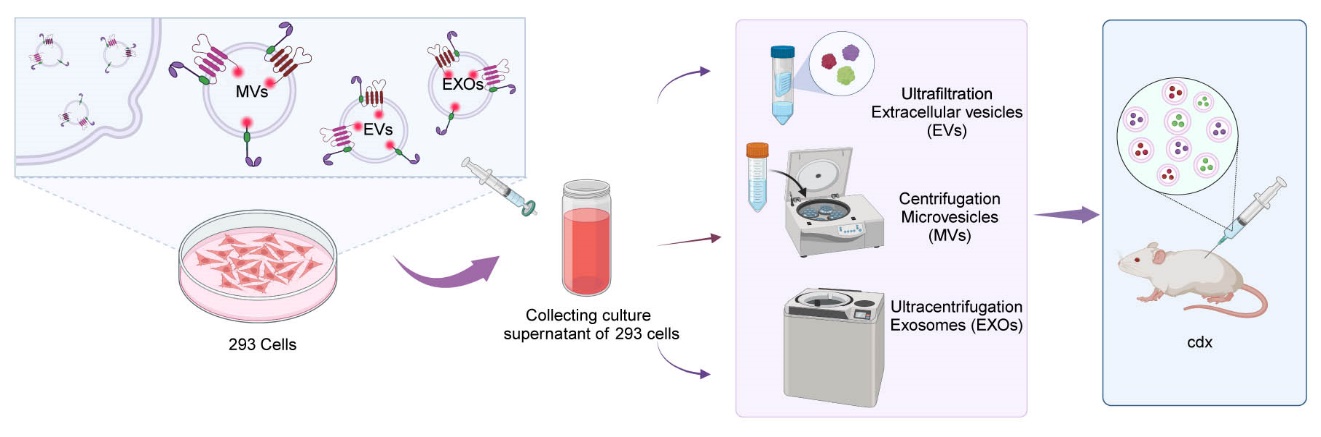


**Supplementary Fig. 17. Preparation of engineered EVs, MVs, and EXOs.** Image created with BioRender.com, used with permission.


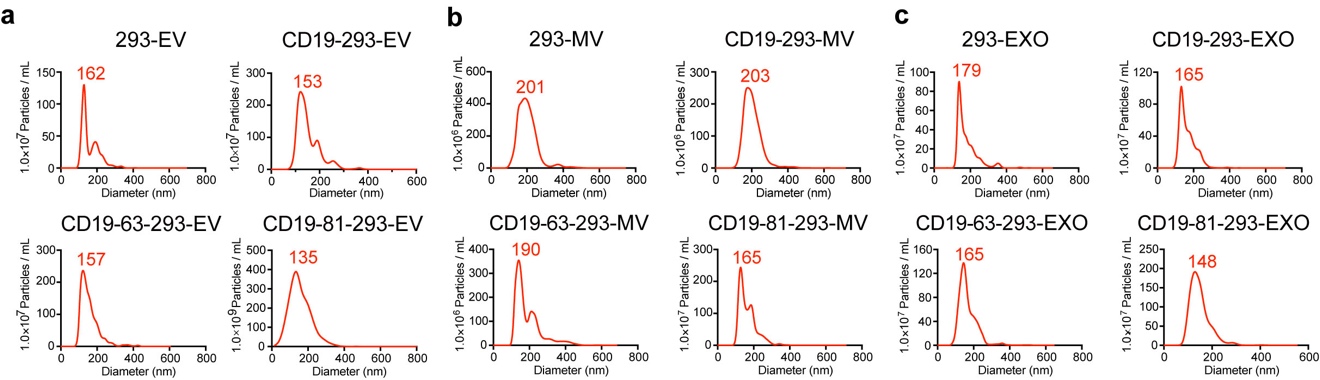


**Supplementary Fig. 18. Characterization of EVs, MVs, and EXOs.** NTA characterization of particle size of **(a)** EVs, **(b)** MVs, and **(c)** EXOs from 293, CD19-293, CD19-63-293, and CD19-81-293 cells. Experiments were performed in biological triplicate.


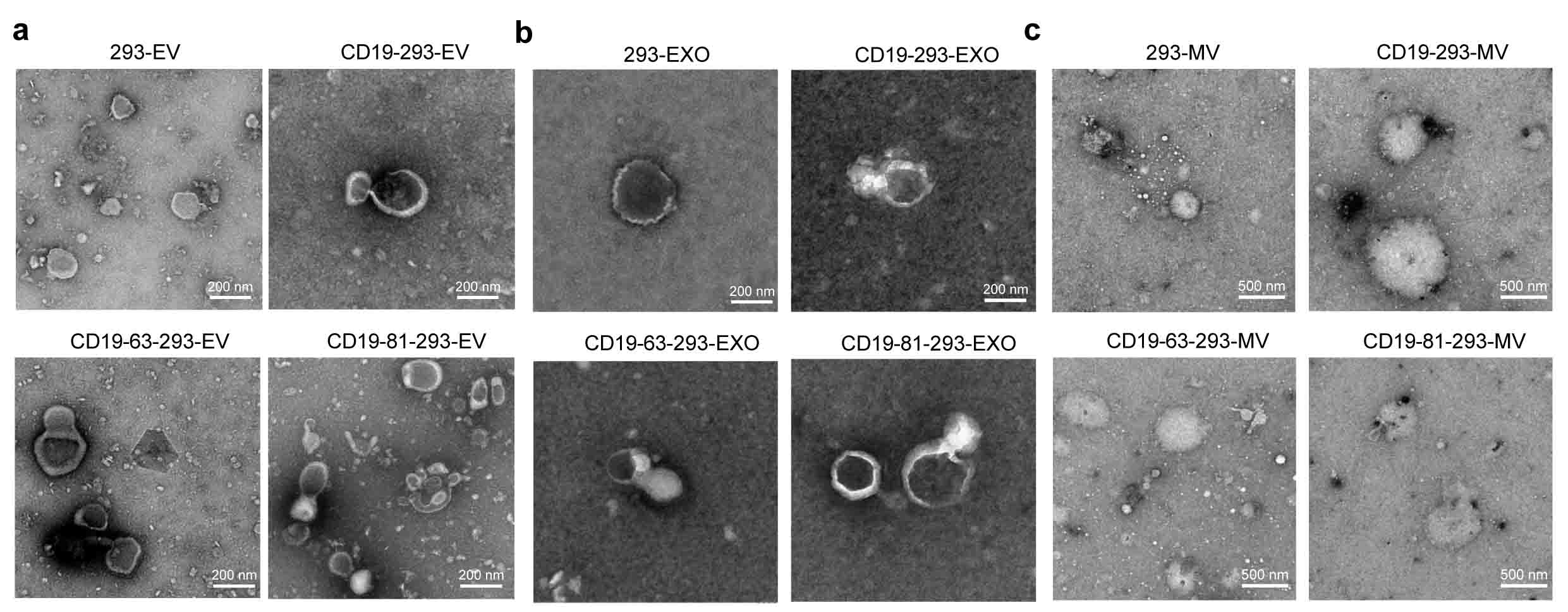


**Supplementary Fig. 19. Characterization of EVs, MVs, and EXOs.** TEM characterization of the morphology **(a)** EVs, **(b)** EXOs, and **(c)** MVs from 293, CD19-293, CD19-63-293, and CD19-81-293 cells. Scale bar: 200–500 nm.


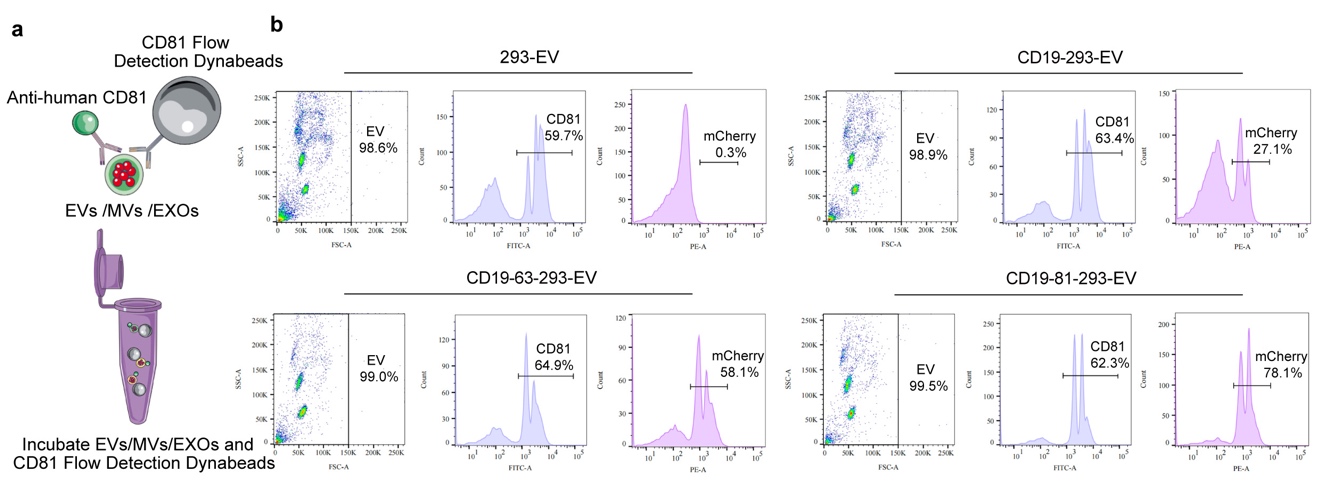


**Supplementary Fig. 20. Generation of EV, MV, and EXO pattern maps using CD81 flow detection dynabeads.** **(a)** Pattern diagram of the CD81-dynabeads flow assay. **(b)** Flow analysis plot of each group of EVs.


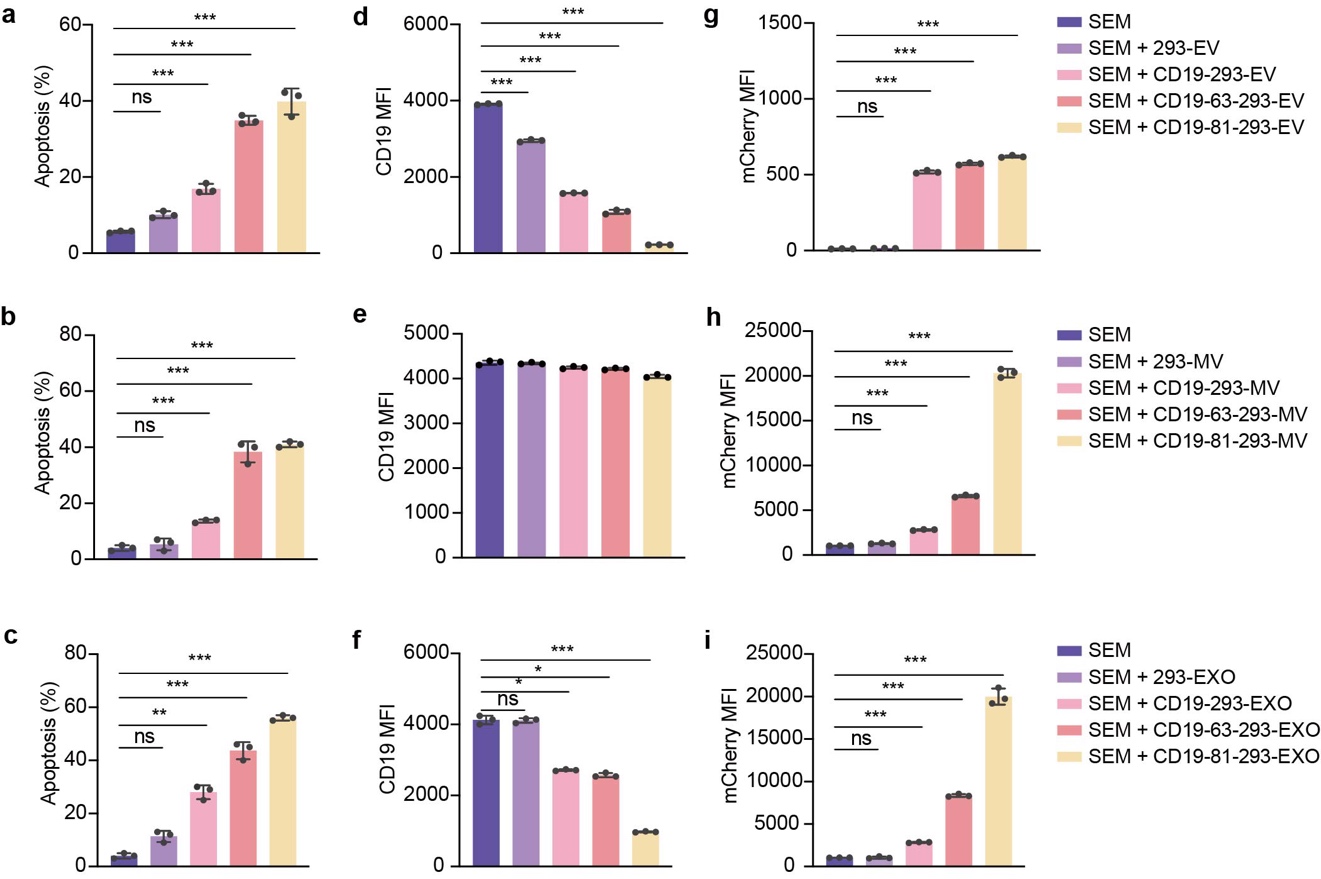


**Supplementary Fig. 21. Apoptosis, CD19 MFI, and vesicle uptake in target cells after treatment with engineered vesicles. (a**-**c)** Target cell apoptosis, **(d-f)** CD19 MFI, and **(g-i)** vesicle uptake in SEM cells after 24 h treatment with **(a, d, g)** EVs, **(d, e, f)** MVs, and **(g, h, i)** EXOs. mCherry-MFI was used to determine the uptake level. The representative result of three independent experiments is shown. Each data point represents the means ± SD (n=3). Statistical analysis was performed using Student’s *t*-test for the unpaired data. Statistical significance: *** *p*<0.001.


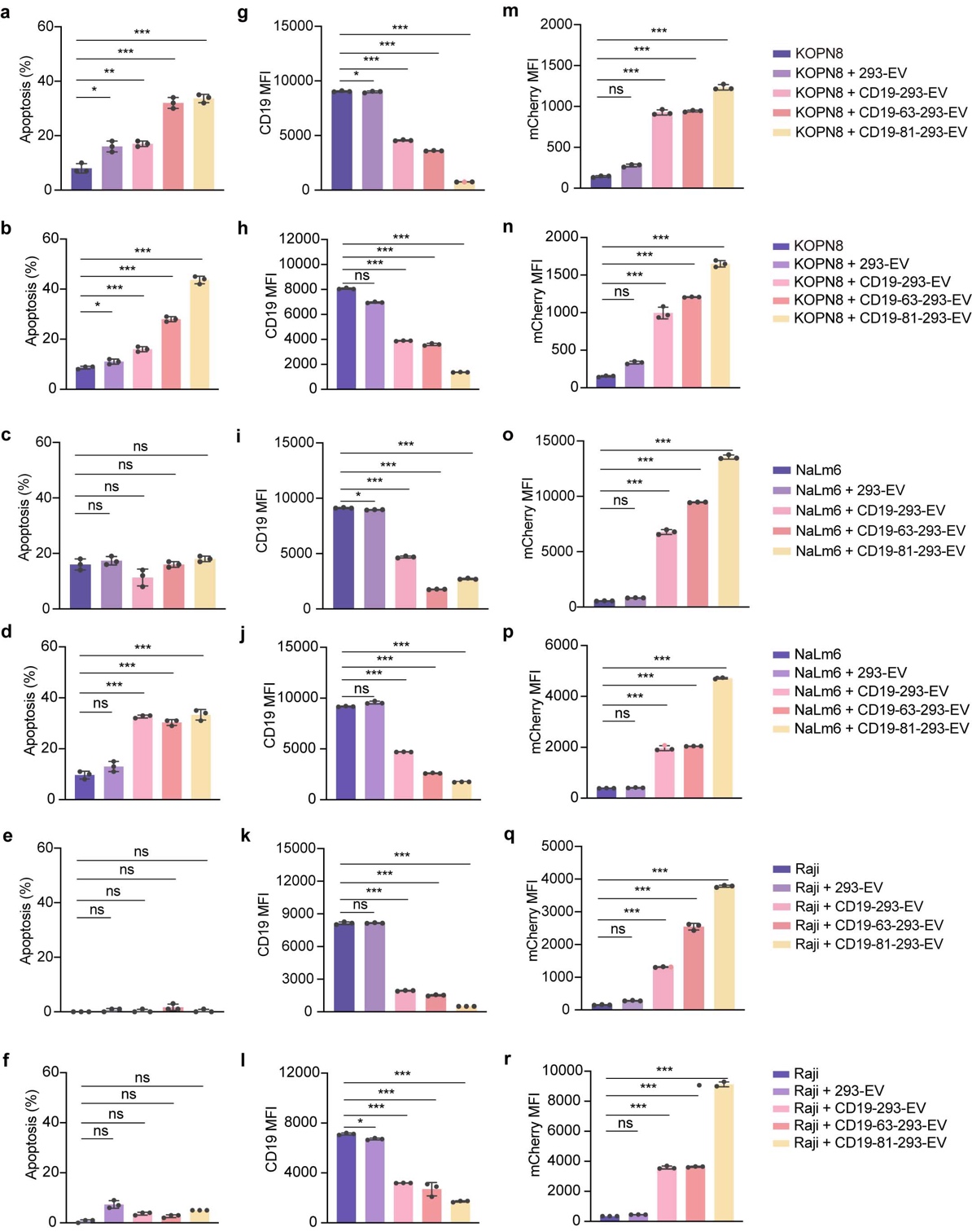


**Supplementary Fig. 22. Apoptosis, CD19 MFI, and vesicle uptake in target cells after treatment with engineered vesicles. (a-f)** Target cell apoptosis assay in **(a-b)** KOPN8, **(c-d)** NaLm6, and **(e-f)** Raji cells treated with engineered EVs for **(a, c, e)** 24 and **(b, d, f)** 48 h. CD19 MFI in **(g-h)** KOPN8, **(i-j)** NaLm6, and **(k-l)** Raji cells treated with engineered EVs for **(g, i, k)** 24 and **(h, j, l)** 48 h. Uptake level in **(m-n)** KOPN8, **(o-p)** NaLm6, and **(q-r)** Raji cells treated with engineered EVs for **(m, o, q)** 24 and **(n, p, r)** 48 h. mCherry-MFI was used to determine the uptake level. The representative result of three independent experiments is shown. Each data point represents the means ± SD (n=3). Statistical analysis was performed using Student’s *t*-test for the unpaired data. Statistical significance: *** *p*<0.001.


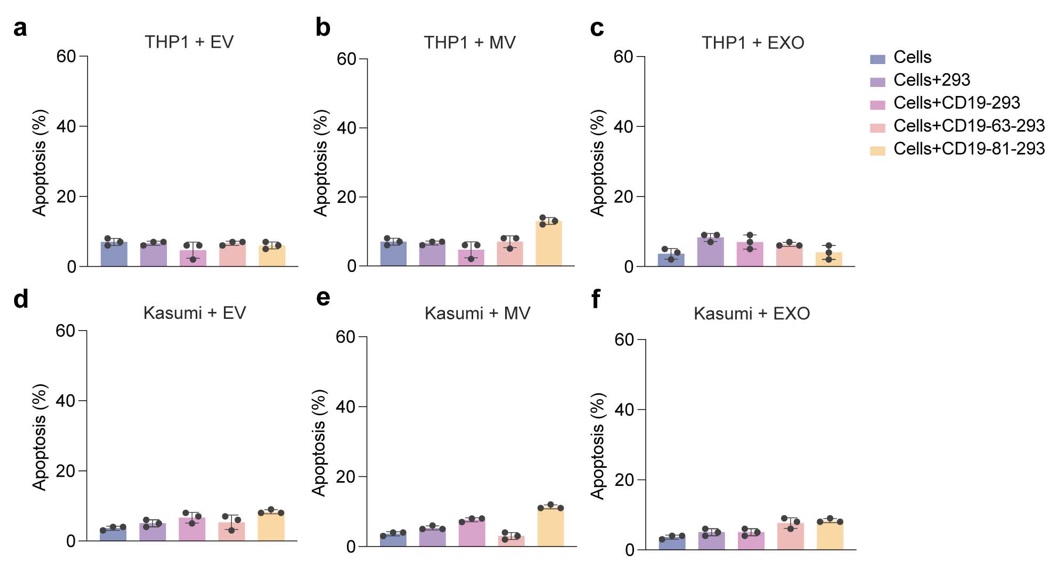


**Supplementary Fig. 23.** **Apoptosis in AML cells treated with engineered vesicles.** Target cell apoptosis assay in **(a-c)** THP1 and **(d-f)** Kasumi-1 cells treated for 48 h with **(a, d)** EVs, **(b, e)** MVs, and **(c, f)** EXOs from 293, CD19-293, CD19-63-293, and CD19-81-293 cells. The representative result of three independent experiments is shown. Each data point represents the means ± SD (n=3)

**
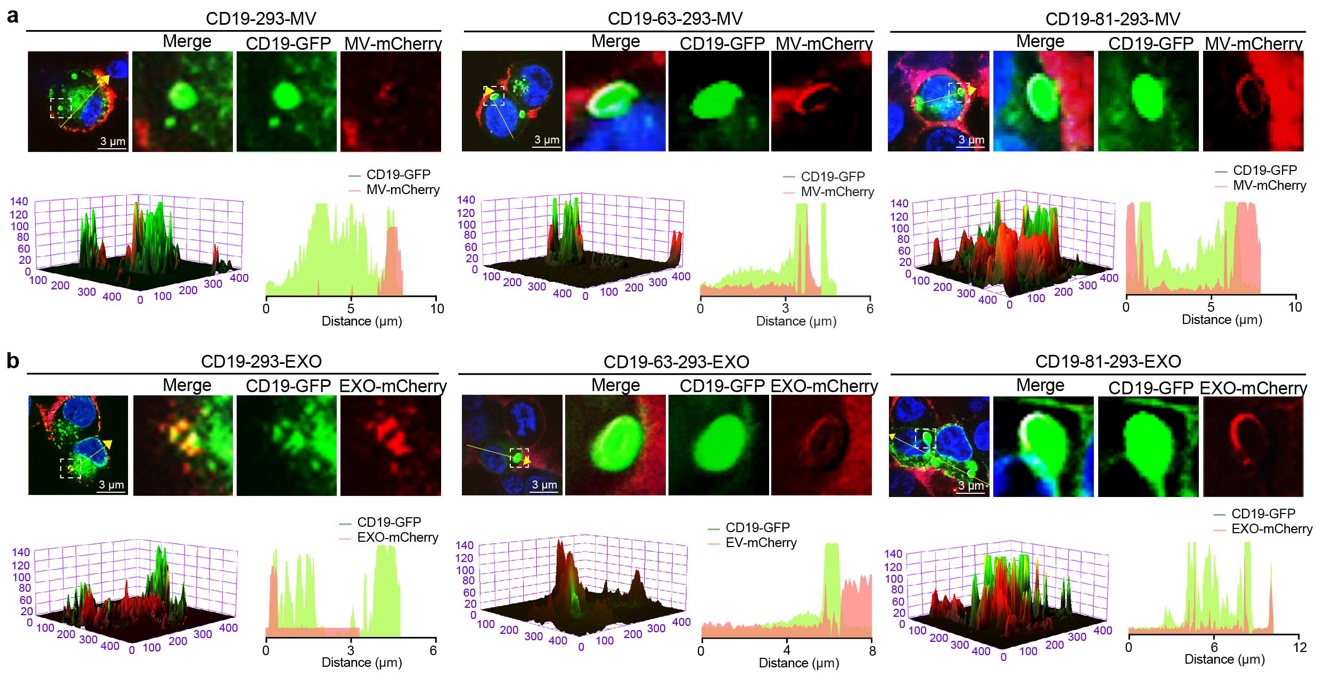
**

**Supplementary Fig. 24. Engineered vesicles with higher abundances of targeting modules induce stronger CD19 endocytosis.** High-resolution confocal images showing the co-localization of CD19 protein and engineered vesicles in 293-CD19-GFP cells after 10-min treatment with **(a)** MVs and **(b)** EXOs, along with 3D surface plots of fluorescence and corresponding fluorescence curves (blue: Hoechst, green: CD19 protein, red: EVs, MVs, and EXOs, yellow: merge). Scale bar: 3 μm.

**
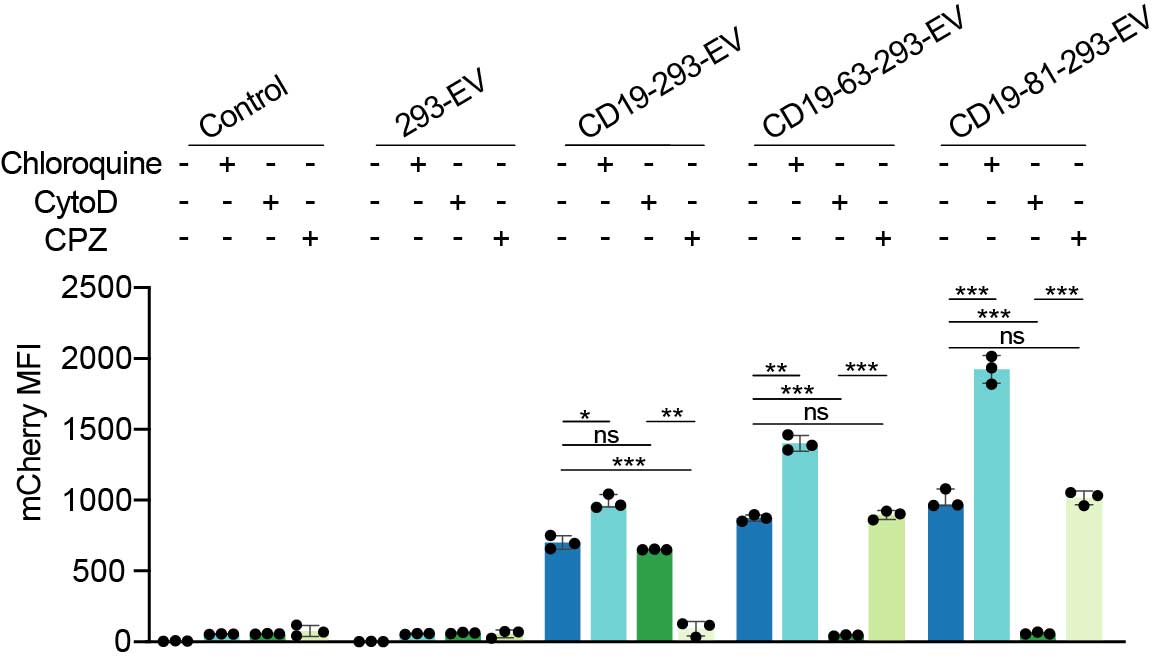
**

**Supplementary Fig. 25. Endocytosed CD19 protein is degraded by lysosomes after EVs trigger the ADE pathway.** Detection of EV uptake level in SEM cells after 48 h treatment with engineered EVs, chloroquine (10 μM), CytoD (10 μM), and chlorpromazine (CPZ, 10μM). PBS was used as the control. The representative result of three independent experiments is shown. Each data point represents the means ± SD (n=3). Statistical analysis was performed using Student’s *t*-test for the unpaired data. Statistical significance: *** *p*<0.001.

**
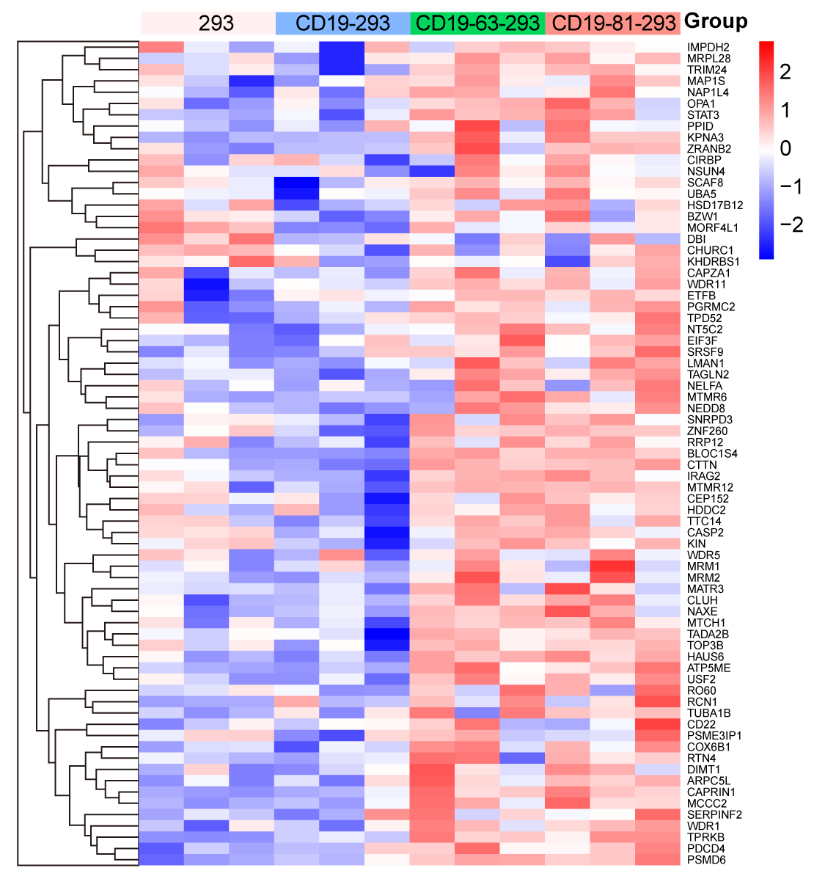
**

**Supplementary Fig. 26. Proteins upregulated in SEM cells after treatment with engineered EVs for 24 h compared with CD19-293-EV.**

**
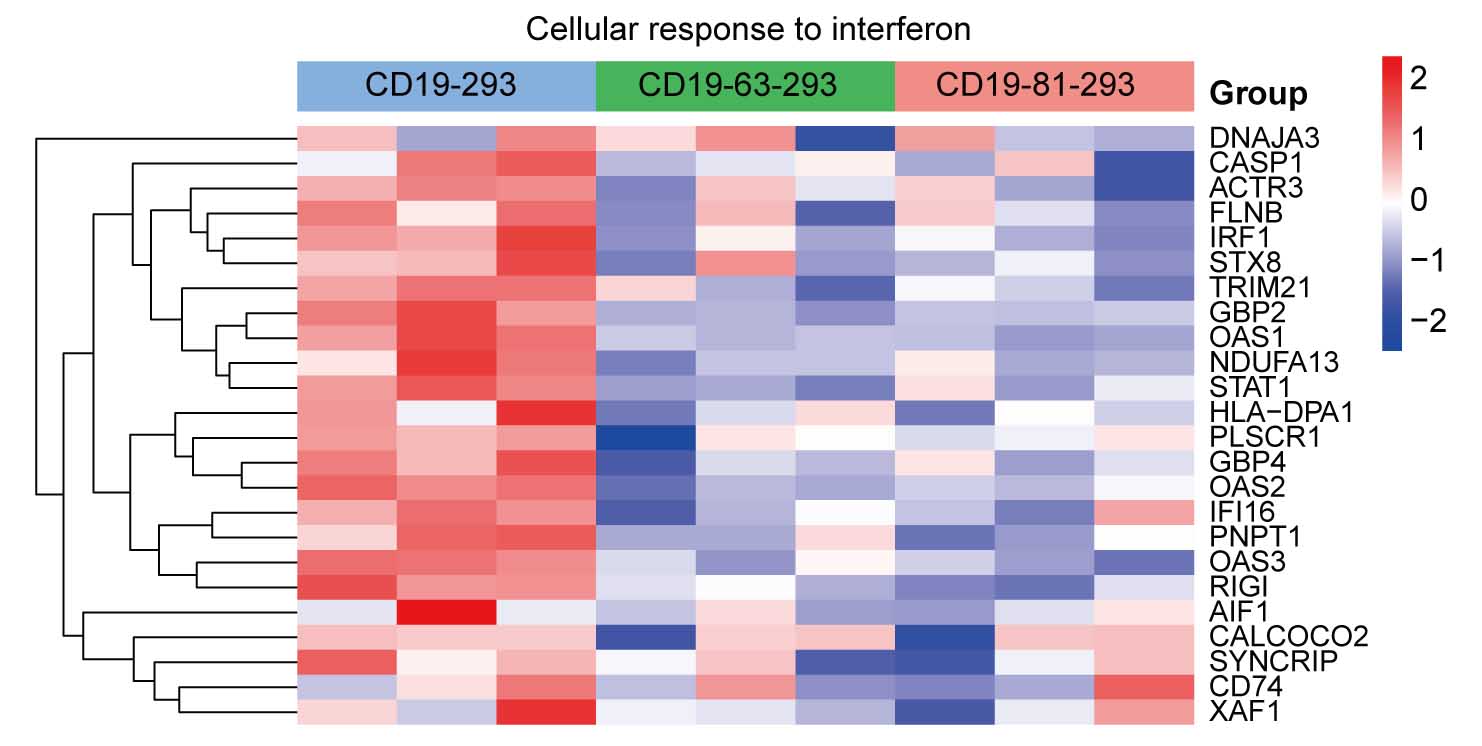
**

**Supplementary Fig. 27.** **Proteomic analysis of SEM cells after treatment with engineered EVs for 24 h.** Heatmap of key pathways. Cellular response to interferon was extracted and visualized in a clear heatmap.

**
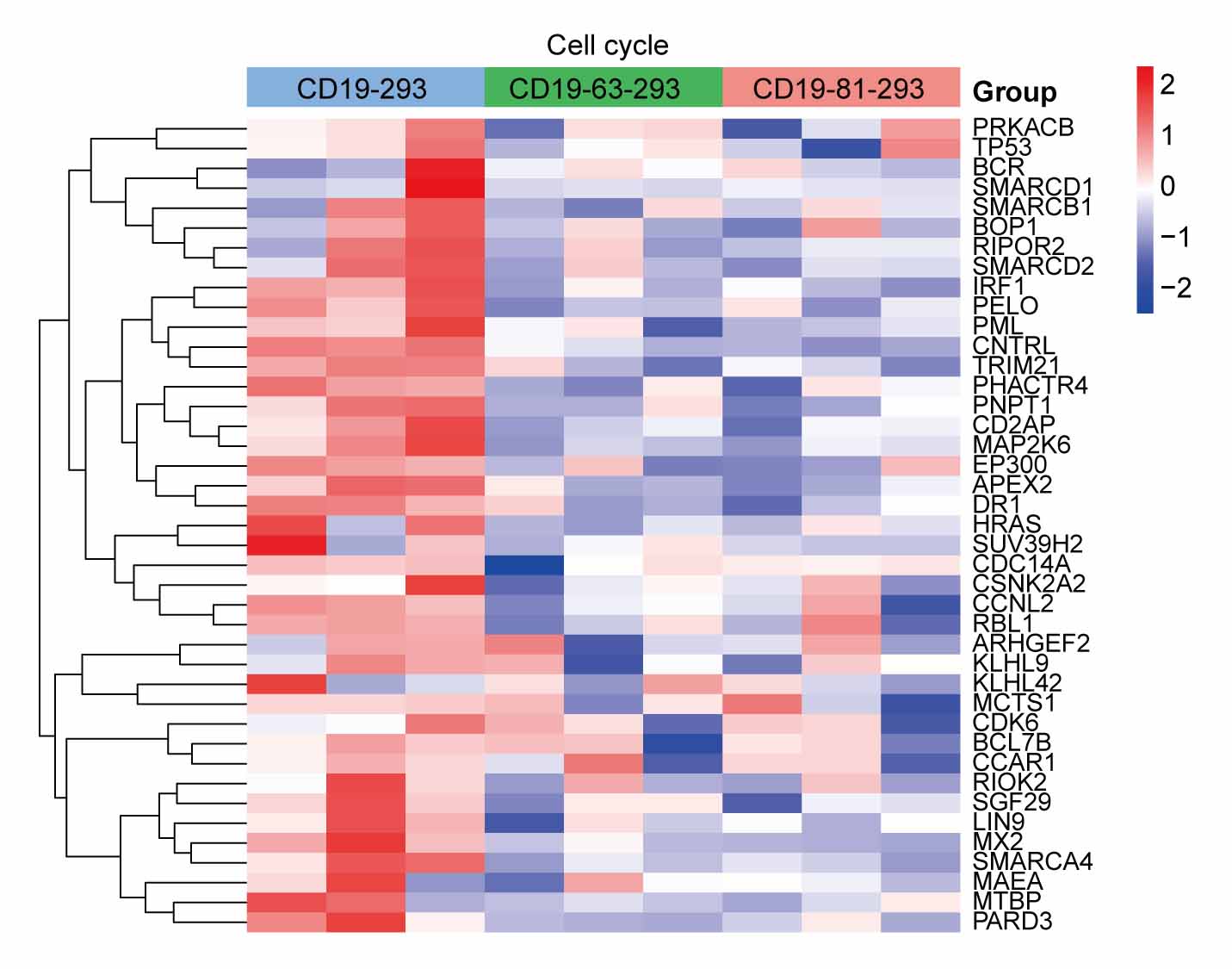
**

**Supplementary Fig. 28. Proteomic analysis of SEM after treatment with engineered EVs for 24 h.** Heatmap of key pathways. The cell cycle was extracted and visualized in a clear heatmap.

**
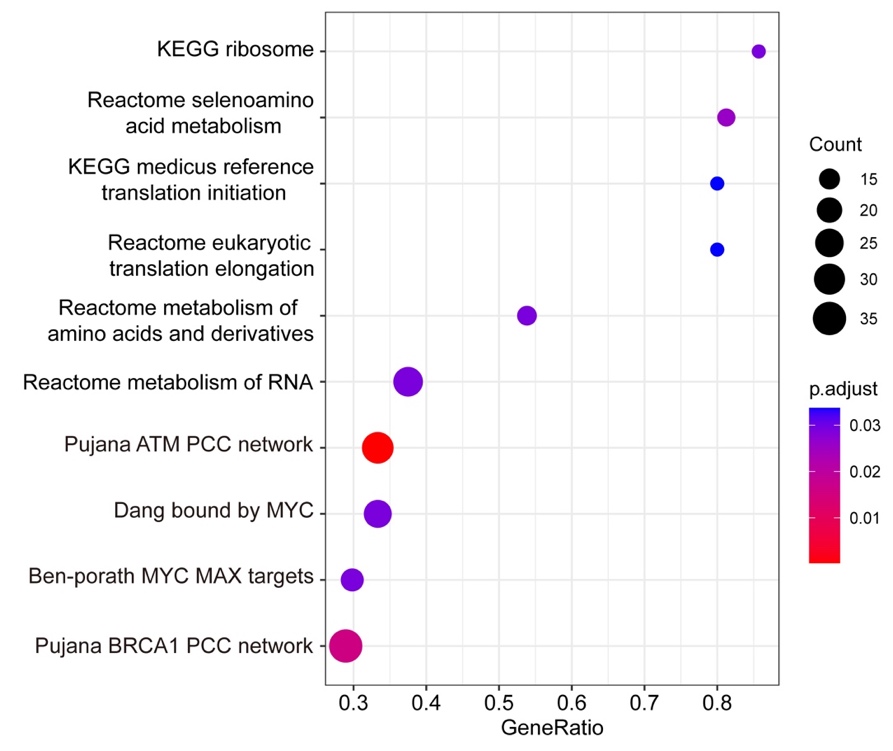
**

**Supplementary Fig. 29. Proteomic analysis of SEM after treatment with engineered EVs for 24 h.** Bubble maps were obtained by GSEA enrichment analysis, showing the enrichment of MYC pathways.

**
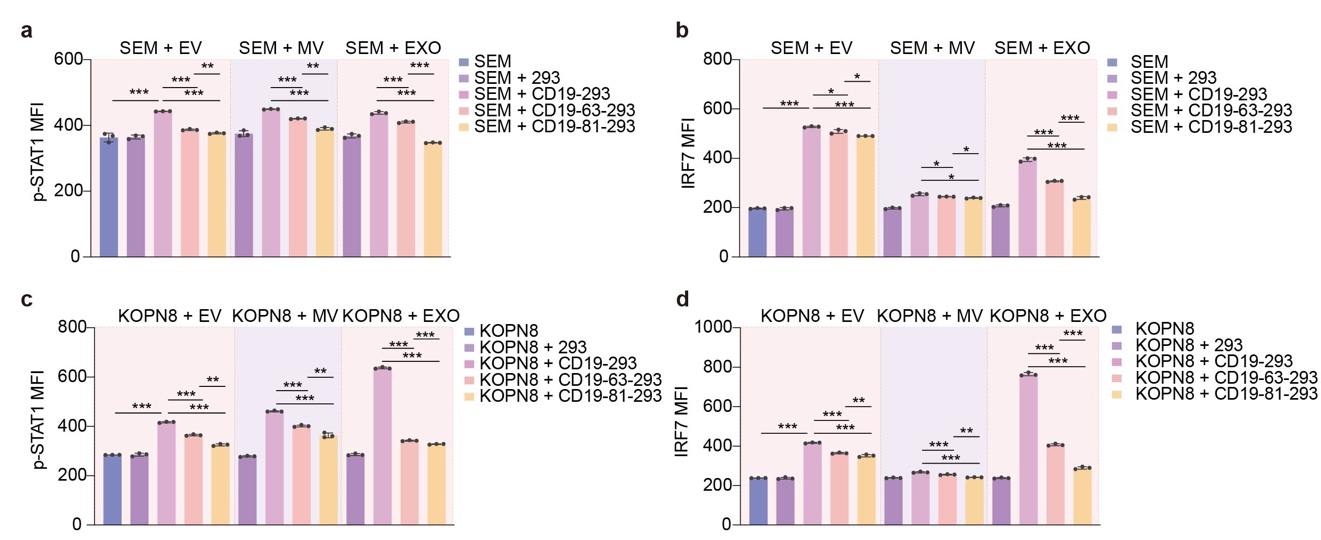
**

**Supplementary Fig. 30.** **p-STAT1 and IRF7 MFI in target cells after treatment with engineered vesicles. (a, b)** SEM and **(c, d)** KOPN8 cells were treated for 4 h with engineered EVs, MVs, and EXOs. Flow cytometry was used to detect **(a, c)** p-STAT1 and **(b, d)** IRF7 MFI of target cells. The representative result of three independent experiments is shown. Each data point represents the means ± SD (n=3). Statistical analysis was performed using Student’s *t*-test for the unpaired data. Statistical significance: *** *p*<0.001.

**
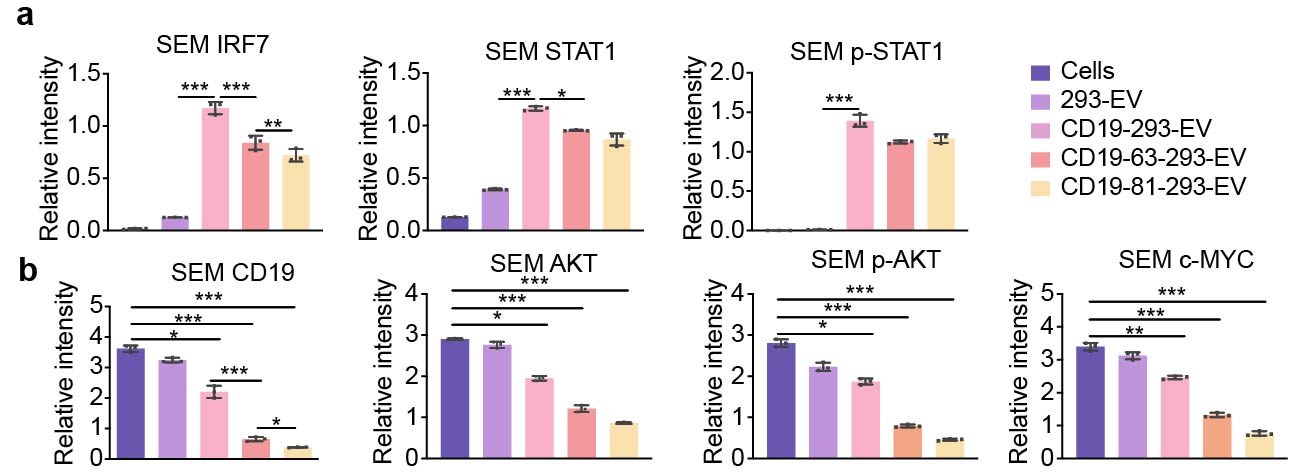
**

**Supplementary Fig. 31.** **Interferon pathway and CD19/PI3K/AKT protein expression in SEM cells after treatment with engineered EVs.** Histogram was the gray analysis of **Figure 5g (a)** and **Figure 5h (b)** bands. The representative result of three independent experiments is shown. Each data point represents the means ± SD (n=3). Statistical analysis was performed using Student’s *t*-test for the unpaired data. Statistical significance: *** *p*<0.001.

**
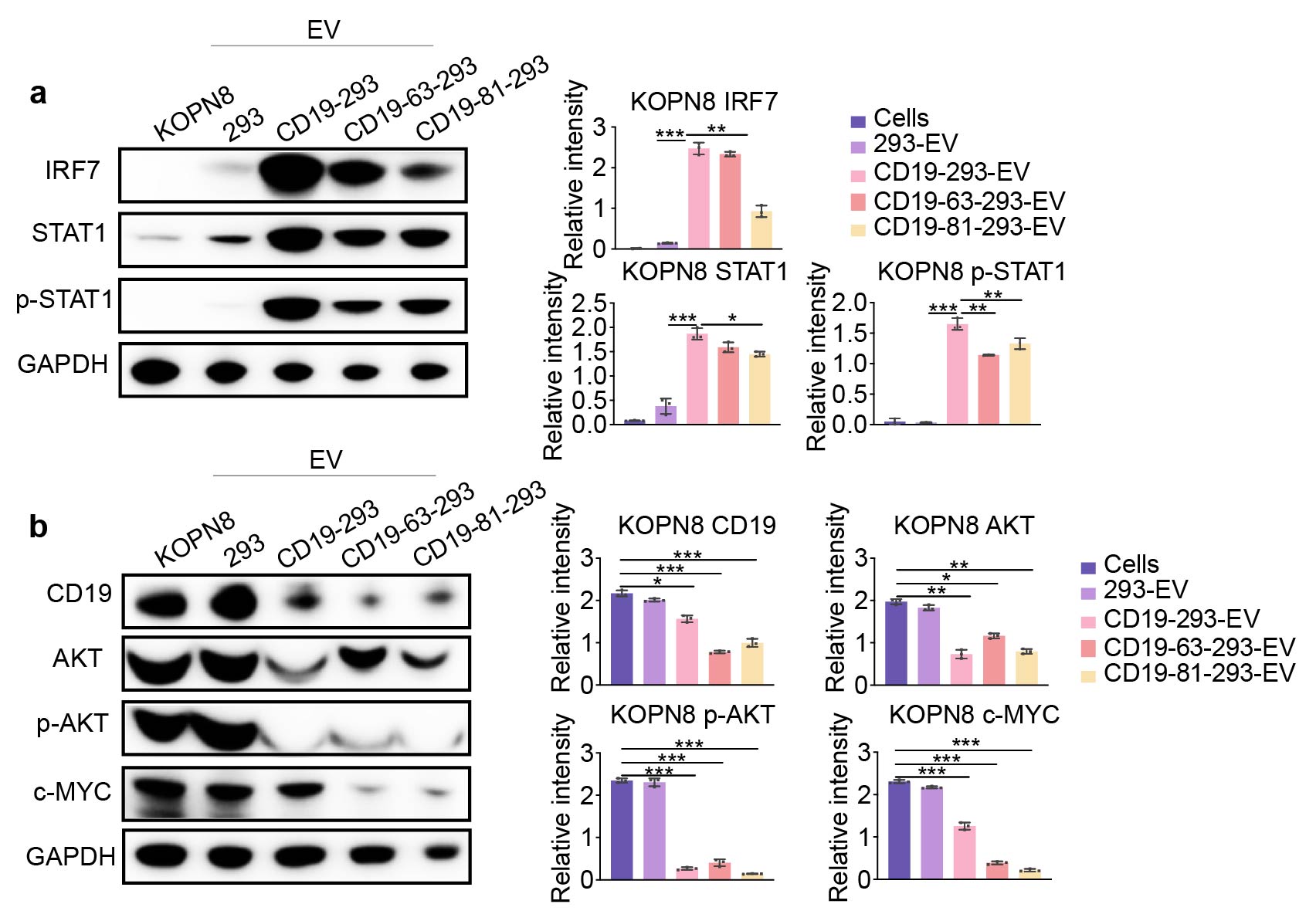
**

**Supplementary Fig. 32.** **Interferon pathway and CD19/PI3K/AKT protein expression in KOPN8 cells after treatment with engineered EVs.** Immunoblots of KOPN8 cells after treatment with EVs for 4 or 24 h. **(a)** Detection of interferon pathway on the target cell surface. **(b)** CD19 and PI3K/AKT/c-MYC protein levels. Histogram is the gray analysis of the bands. The representative result of three independent experiments is shown. Each data point represents the means ± SD (n=3). Statistical analysis was performed using Student’s *t*-test for the unpaired data. Statistical significance: *** *p*<0.001.


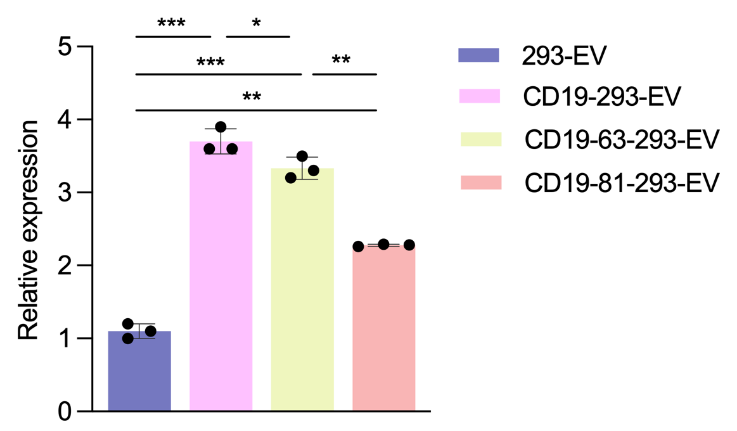


**Supplementary Fig. 33. The qPCR data demonstrate that engineered EVs treatment induces IFN response-related genes (*IRF7*) in SEM cells.** 293-EV as a negative control. The representative result of three independent experiments is shown. Each data point represents the means ± SD (n=3). Statistical analysis was performed using the Student’s t-test for the unpaired data. Statistical significance: *** p<0.001.

**
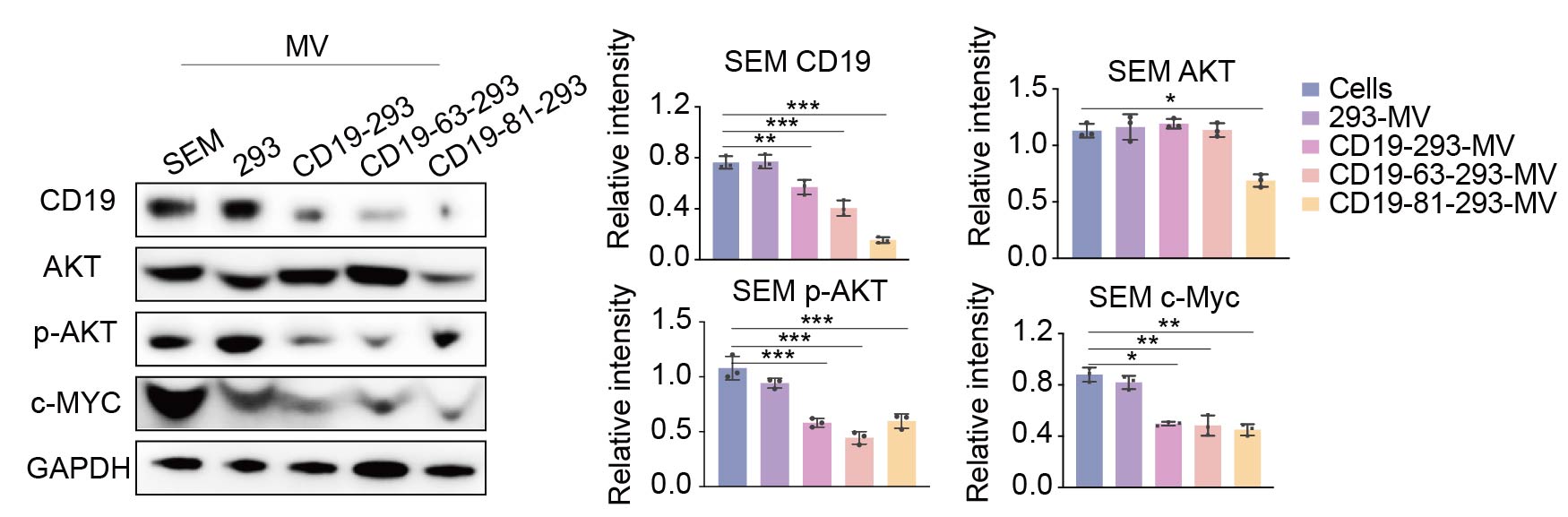
**

**Supplementary Fig. 34.** **CD19/PI3K/AKT protein expression in SEM cells after treatment with engineered MVs.** Immunoblots of SEM cells after 24 h treatment with engineered MVs. Altered CD19 protein level on the surface of SEM cells, and effects of the PI3K/AKT pathway. Histogram is the gray analysis of the bands. The representative result of three independent experiments is shown. Each data point represents the means ± SD (n=3). Statistical analysis was performed using Student’s *t*-test for the unpaired data. Statistical significance: *** *p*<0.001.

**
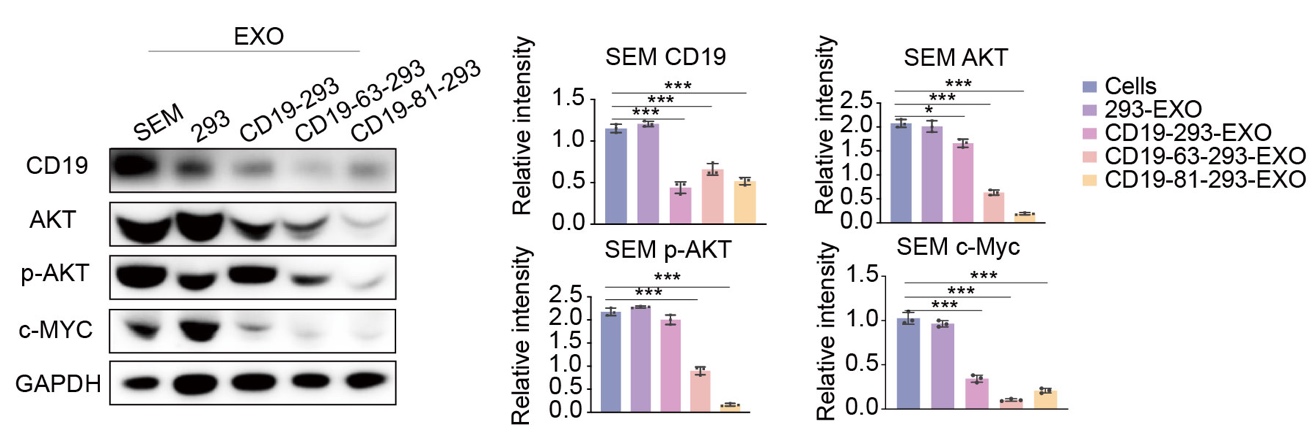
**

**Supplementary Fig. 35. CD19/PI3K/AKT protein expression in SEM cells after treatment with engineered EXOs.** Immunoblots of SEM cells after 24 h treatment with engineered EXOs. Altered CD19 protein level on the surface of SEM cells, and effects of the PI3K/AKT pathway. Histogram is the gray analysis of the bands. The representative result of three independent experiments is shown. Each data point represents the means ± SD (n=3). Statistical analysis was performed using Student’s *t*-test for the unpaired data. Statistical significance: *** *p*<0.001.

**
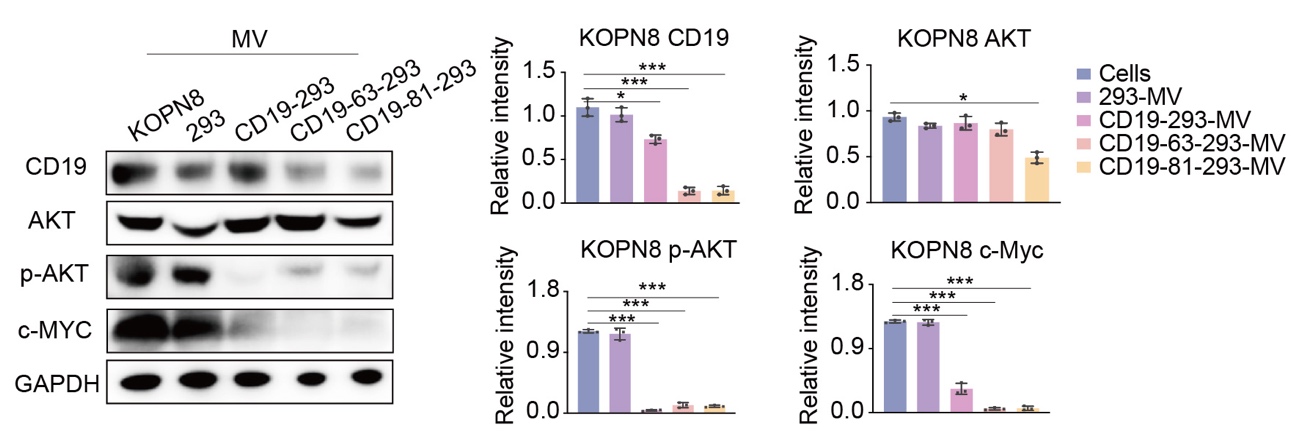
**

**Supplementary Fig. 36. CD19/PI3K/AKT protein expression in KOPN8 cells after treatment with engineered MVs.** Immunoblots of KOPN8 cells after 24 h treatment with engineered MVs. Histogram is the gray analysis of the bands. The representative result of three independent experiments is shown. Each data point represents the means ± SD (n=3). Statistical analysis was performed using Student’s *t*-test for the unpaired data. Statistical significance: *** *p*<0.001.

**
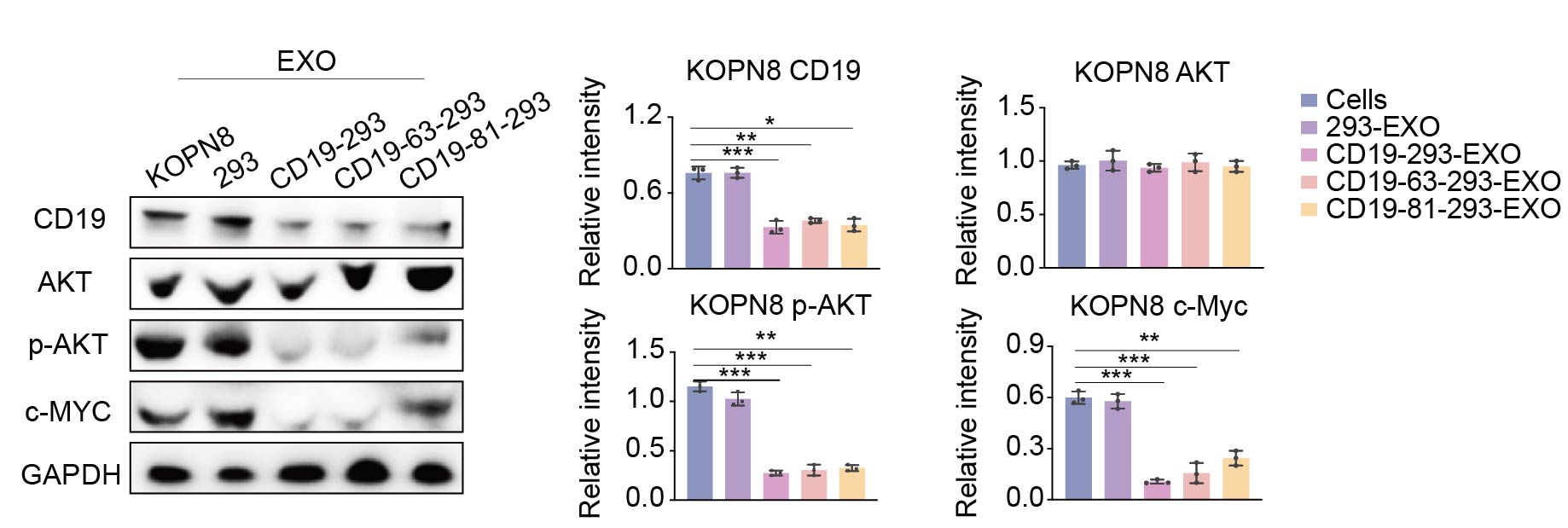
**

**Supplementary Fig. 37. CD19/PI3K/AKT protein expression in KOPN8 cells after treatment with engineered EXOs.** Immunoblots of KOPN8 cells after 24 h treatment with engineered EXOs. Histogram is the gray analysis of the bands. The representative result of three independent experiments is shown. Each data point represents the means ± SD (n=3). Statistical analysis was performed using Student’s *t*-test for the unpaired data. Statistical significance: *** *p*<0.001.

**
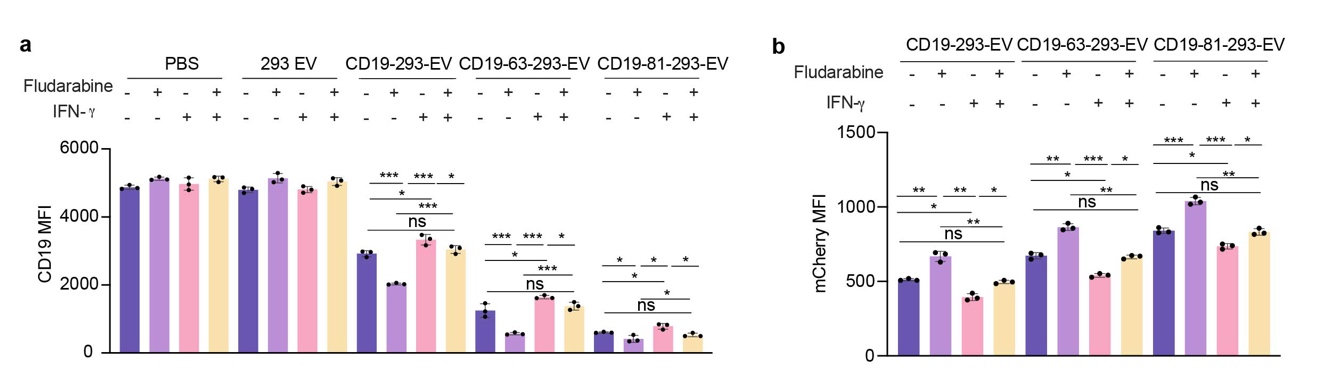
**

**Supplementary Fig. 38. Detection of CD19 MFI and EVs uptake level in SEM cells after treated with EVs under different drug conditions. a-b.** CD19 MFI **(a)** and EVs uptake level **(b)** of SEM after treated with EVs, fludarabine (1 μM) and IFN-γ (5 ng) after 24 hours. The representative result of three independent experiments is shown. means ± SD. n=3. Statistical analysis was performed using the Student’s t-test for the unpaired data. Statistical significance: ***p < 0.001.

**
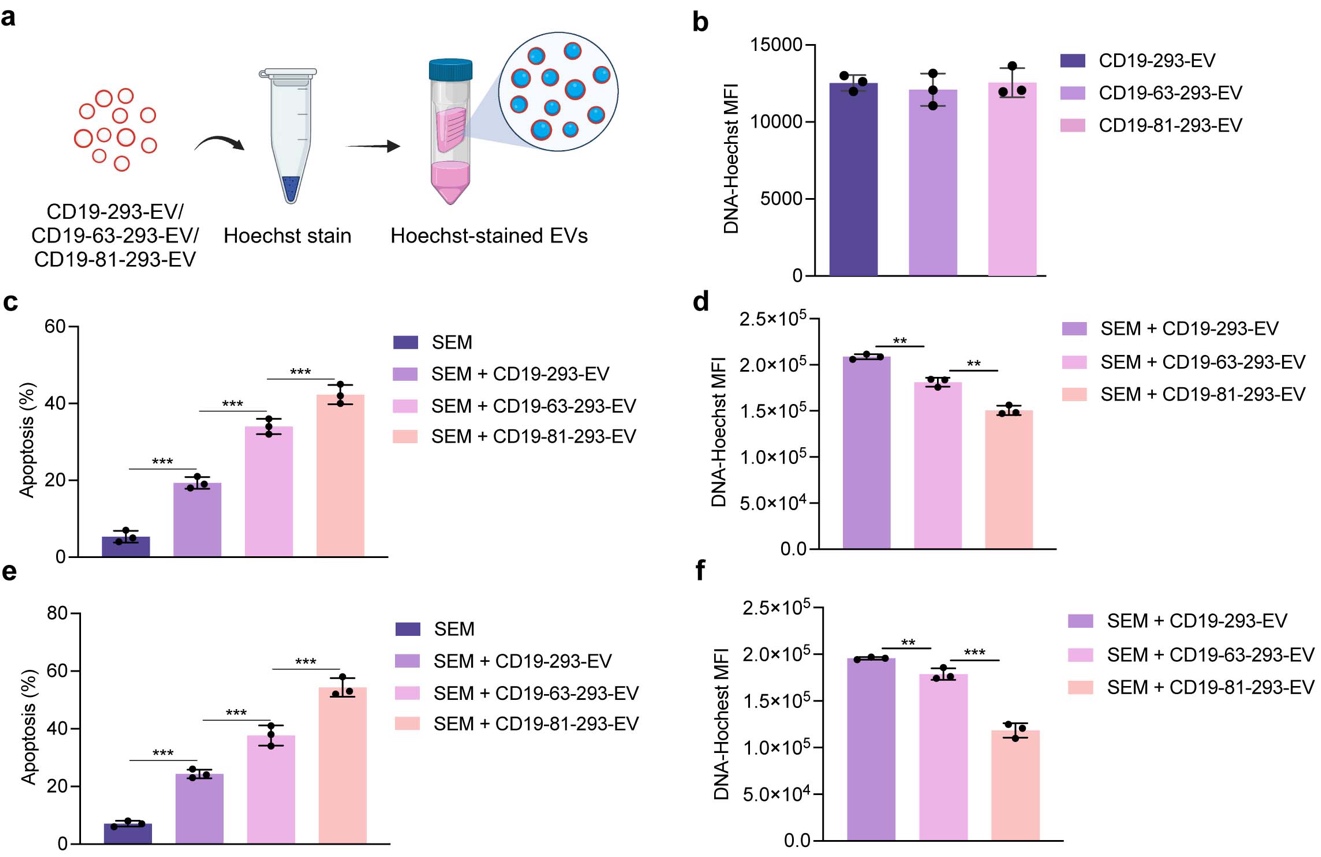
**

**Supplementary Fig. 39. Changes in EV-DNA in SEM cells** **following treatment with Hoechst-stained EVs.** **(a)** Schematic diagram of Hoechst-stained EVs. **(b)** CD81-Dynabeads adsorbed EVs were stained with Hoechst, and the average fluorescence was analyzed by flow cytometry. SEM cells were treated with Hoechst-stained EVs for **(c**-**d)** 24 and **(e-f)** 48 h. **(c, e)** SEM apoptosis and **(d, f)** DNA-Hoechst MFI were detected by flow cytometry. The representative result of three independent experiments is shown. Each data point represents the means ± SD (n=3). Statistical analysis was performed using Student’s *t*-test for the unpaired data. Statistical significance: *** *p*<0.001. Image created with BioRender.com, used with permission.


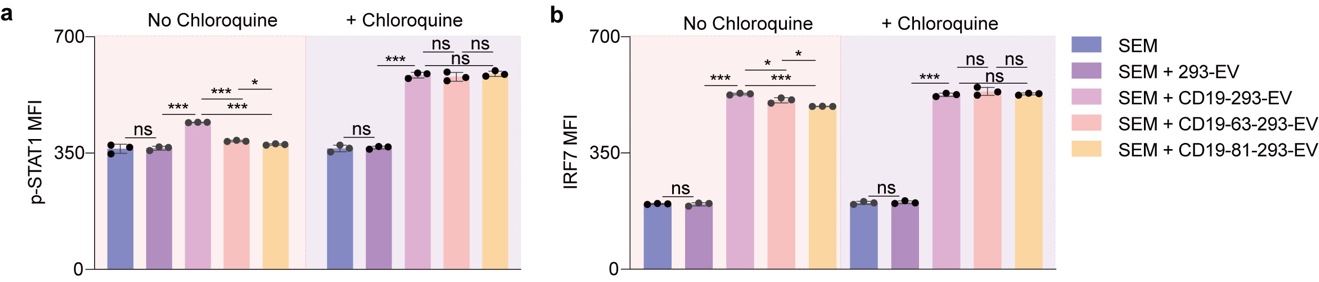


**Supplementary Fig. 40.** **p-STAT1 and IRF7 MFI in SEM cells after treatment with engineered EVs and/or chloroquine.** SEM cells were treated for 4 h with engineered EVs and/or chloroquine (10 μM). **(a)** p-STAT1 and **(b)** IRF7 MFI of target cells were detected by flow cytometry. The representative result of three independent experiments is shown. Each data point represents the means ± SD (n=3). Statistical analysis was performed using the Student’s *t*-test for the unpaired data. Statistical significance: *** *p*<0.001


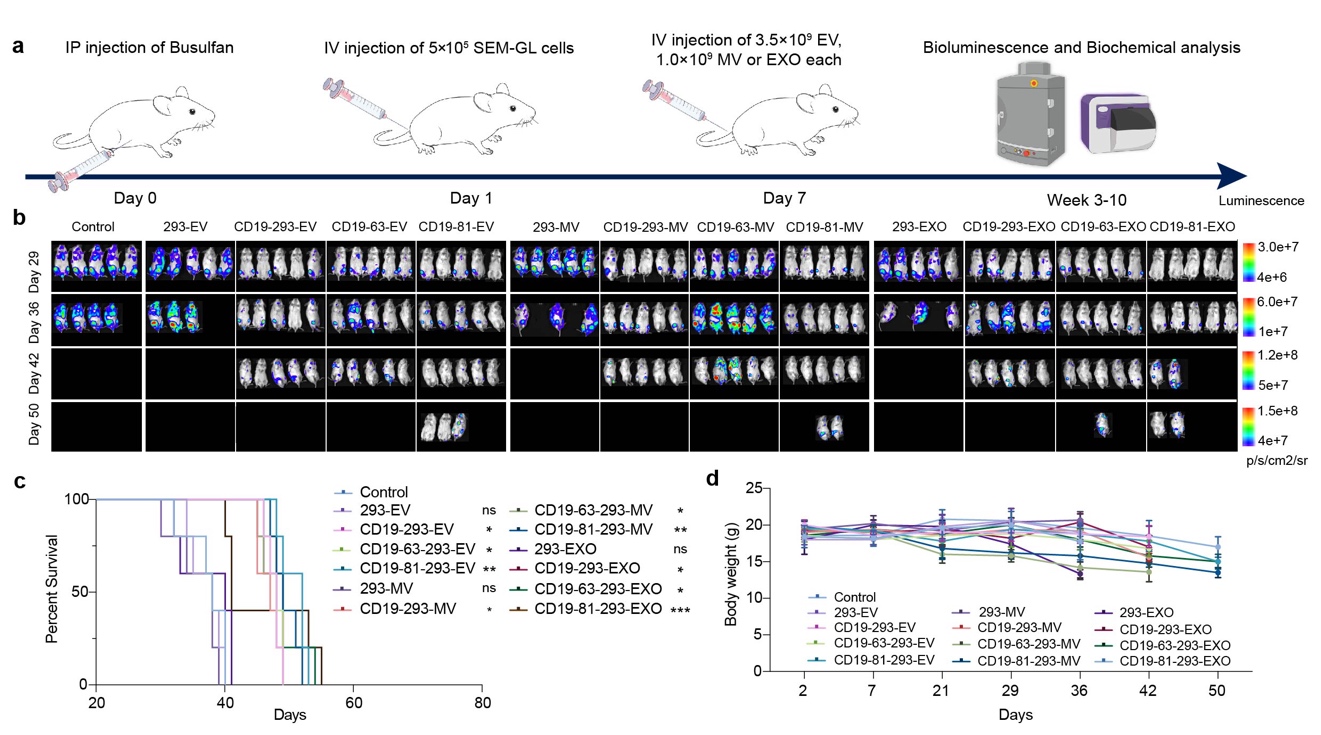


**Supplementary Fig. 41. *In vivo* anti-tumor capacity and biosafety evaluation.** **(a)** Administration schedule of CD19-293-EV/MV/EXO, CD19-63-293-EV/MV/EXO, and CD19-81-293-EV/MV/EXO treatments. **(b)** NOD/SCID mice (n=5) were transplanted with SEM-Luc-GFP cells and treated with engineered EVs. Mice underwent imaging on the indicated days after xenografting to assess leukemia progression. **(c)** Percent survival of **(b)**. CD19-81-293-EXO-treated group showed prolonged survival compared to the control (log-rank Mantel-Cox test). **(d)** Body weight of mice described in **(b)**. Each data point represents the mean ± SD (n=5).


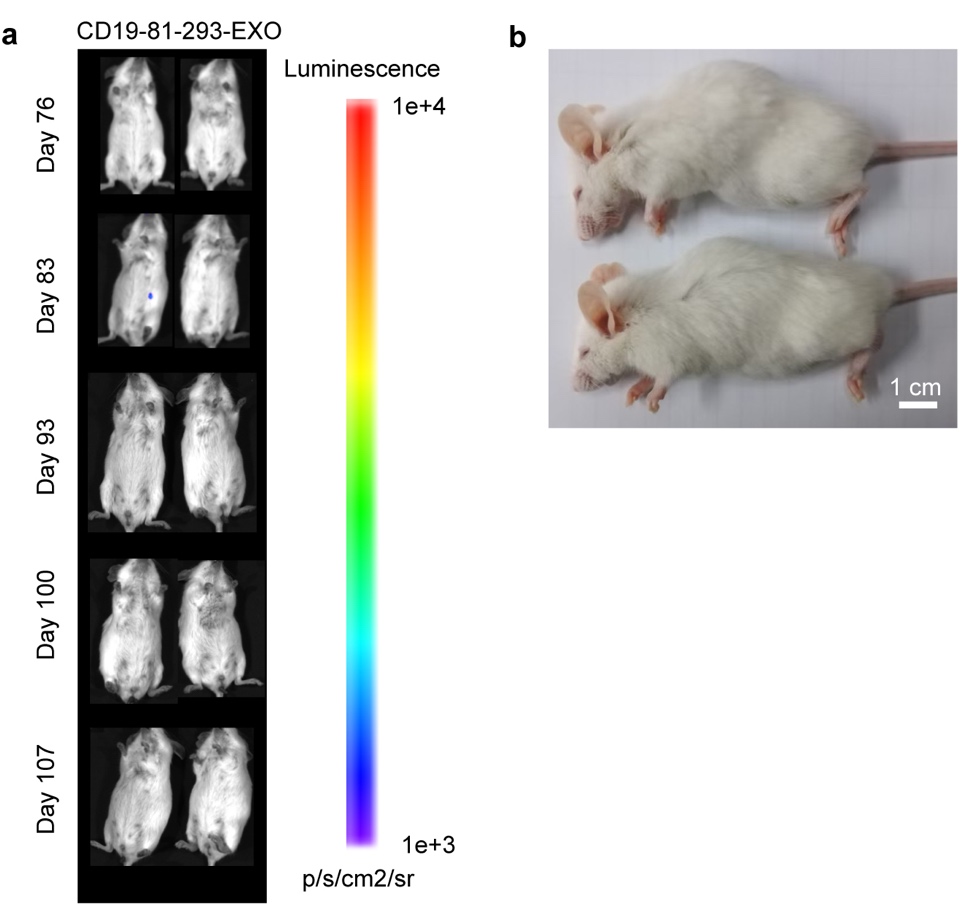


**Supplementary Fig. 42. *In vivo* anti-tumor capacity**. **(a)** NOD/SCID mice transplanted with luciferase-expressing SEM cells were treated with CD19-81-293-EXO until day 107. **(b)** Mice were imaged on the indicated days after xenografting to assess for leukemia progression. Scale bar: 1 cm.


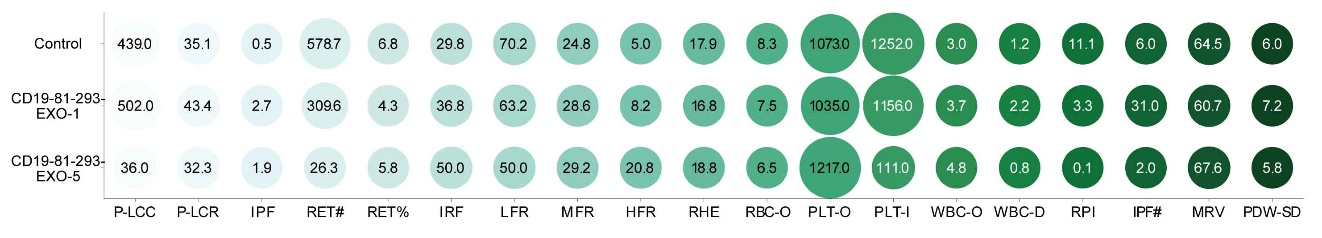


**Supplementary Fig. 43.** **Routine blood analysis.** Routine blood analyses performed on healthy, CD19-81-293-EXO-1, and CD19-81-293-EXO-5 mice.


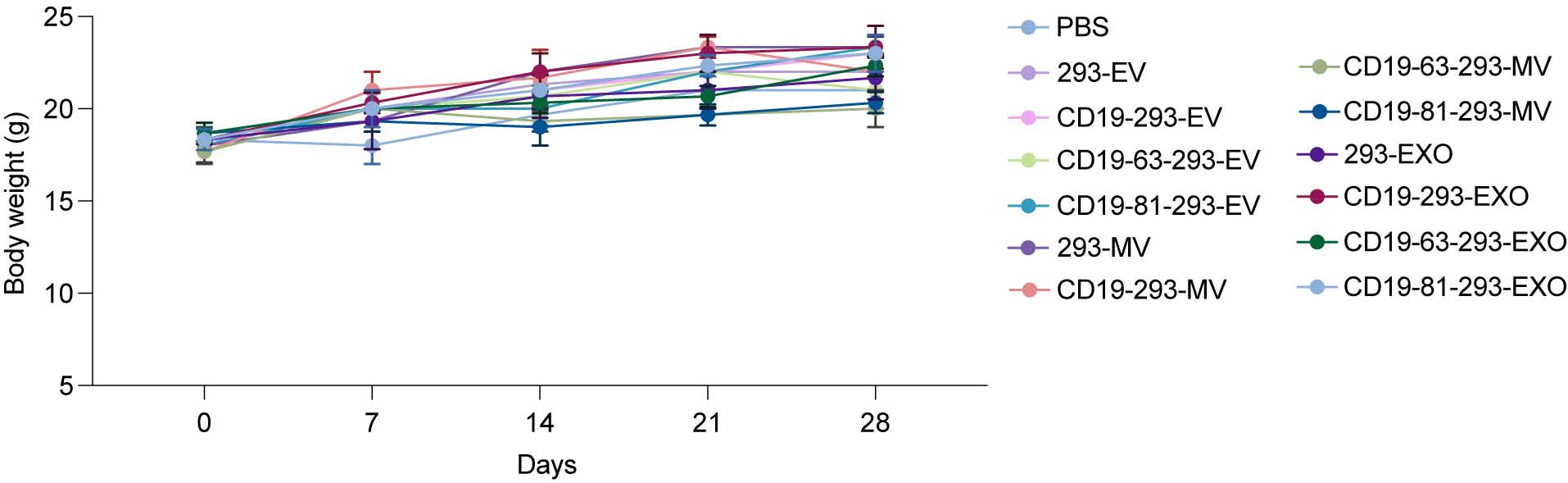


**Supplementary Fig. 44. Body weight changes in NOD/SCID mice 30 days after injection of engineered vesicles.** Each data point represents the mean ± SD (n=3). PBS was used as the control.

Table S1. List of primers used.

Quantitative PCR

| IRF7a-fwd | GCTGGACGTGACCATCATGTA |
| --- | --- |
| IRF7a-rev | GGGCCGTATAGGAACGTGC |
| GAPDH-F | ACAACTTTGGTATCGTGGAAGG |
| GAPDH-R | GCCATCACGCCACAGTTTC |
